# Supplementary material for: A zeolitic vanadotungstate family with structural diversity and ultrahigh porosity for catalysis
Source: Nat Commun. 2018 Sep 17;9:3789. doi: 10.1038/s41467-018-06274-2 (PMC6141569; doi:10.1038/s41467-018-06274-2)
Supplement: Supplementary file 1 — Supplementary Information [file 41467_2018_6274_MOESM1_ESM.pdf]

A zeolitic vanadotungstate family with structural diversity and ultrahigh porosity for catalysis

Zhang et al.

Supplementary Figures

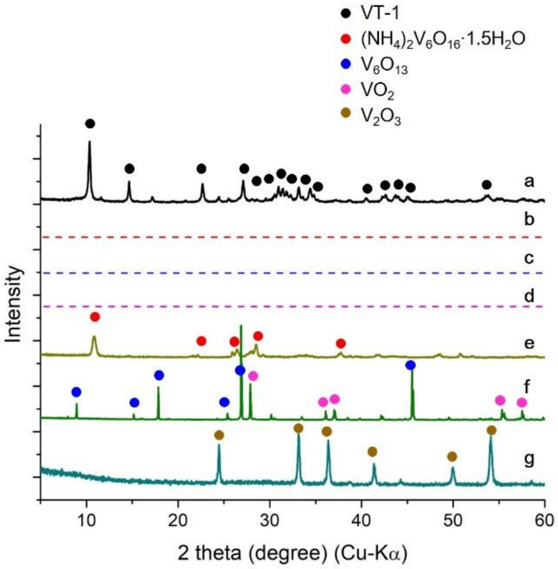

Supplementary Figure 1. XRD patterns of the materials prepared using different V source. (**a**) $VOSO_4$, (**b**) without V, (**c**) NH_4VO_3, (**d**) $NaVO_3$, (**e**) V_2O_5, (**f**) VO_2, and (**g**) V_2O_3, dashed: no solid was obtained.

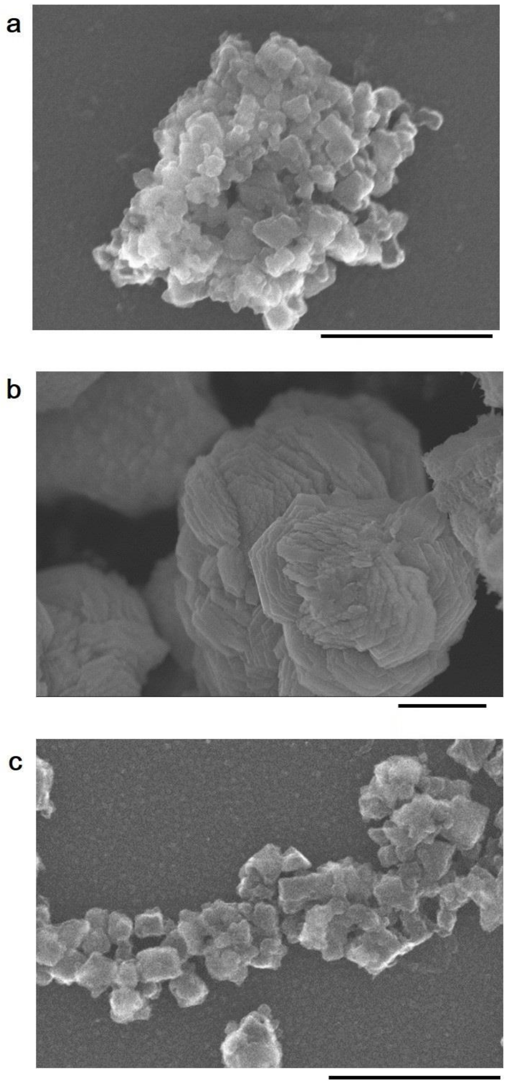

Supplementary Figure 2. SEM images. (**a**) **VT-1**, scale bar: 500 nm, (**b**) **VT-5**, scale bar: 500 nm, and (**c**) **Cs-VT-1**, scale bar: 500 nm.

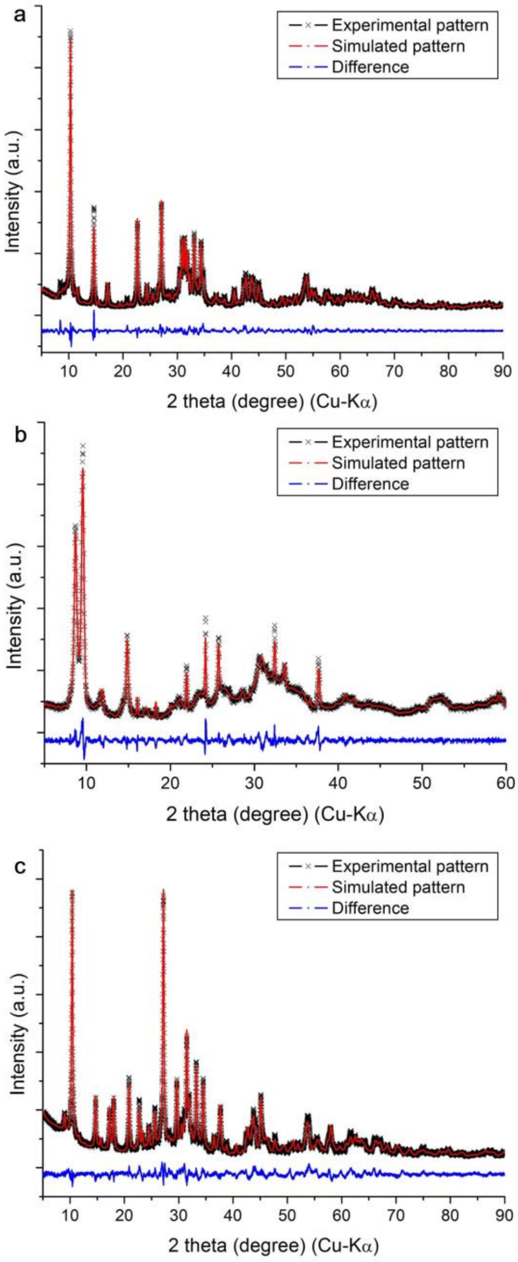

Supplementary Figure 3. Comparison of the experimental XRD patterns with the simulated XRD patterns using the Rietveld method. (**a**) **VT-1**, (**b**) **VT-5**, and (**c**) **Cs-VT-1**.

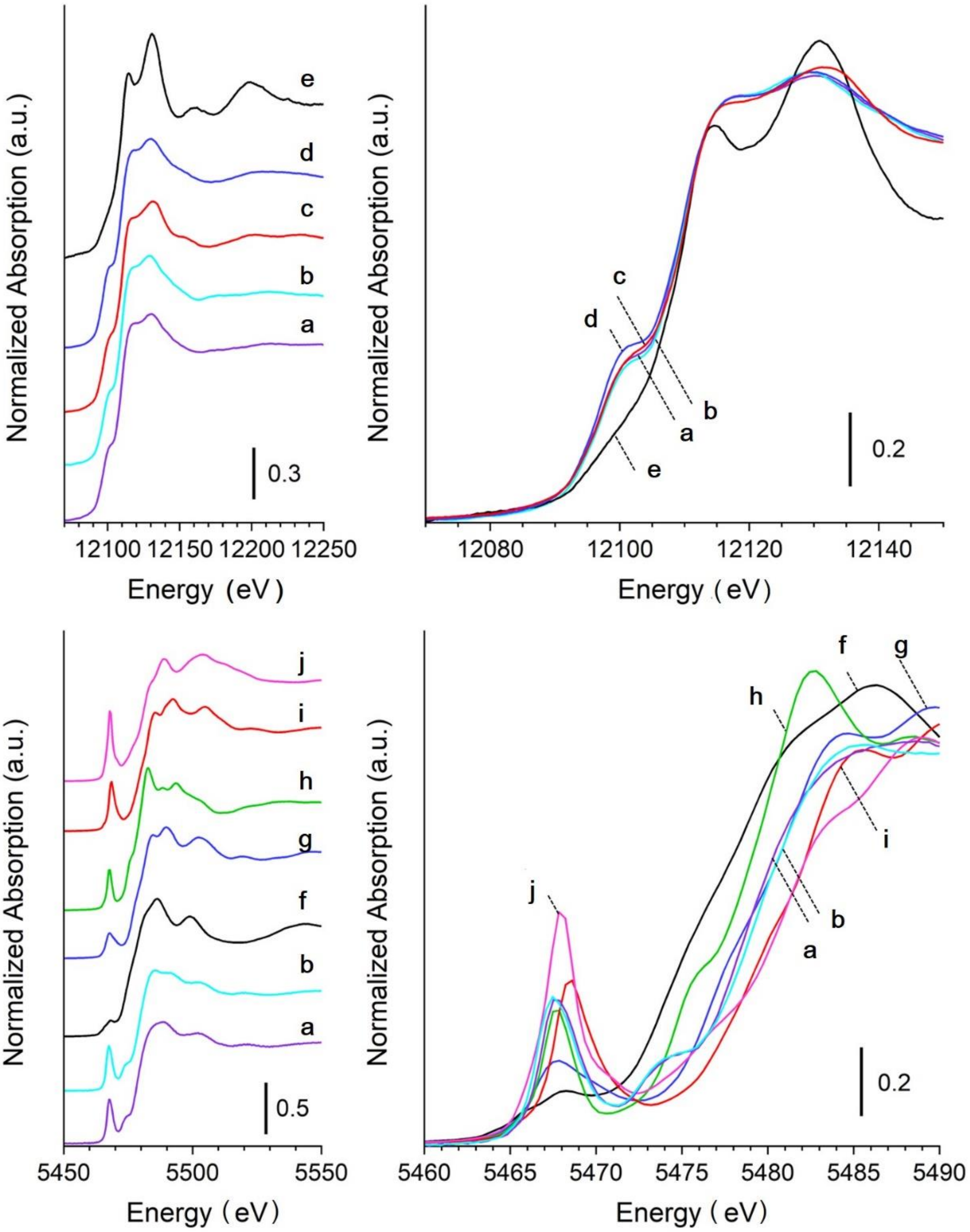

Supplementary Figure 4. XANES spectra of the W L_1-edge and V K-edge. W L_1-edge of (**a**) **VT-1**, (**b**) **VT-5**, (**c**) WO_3, (**d**) Na_2WO_4, and (**e**) Ba_2NiWO_6, V K-edge of (**f**) V_2O_3, (**g**) VO_2, (**h**) α-$VOSO_4$, (**i**) V_2O_5, and (**j**) $NaVO_3$. A vertical offset was added for clarity. The right figures are magnifications of the figures of the left.

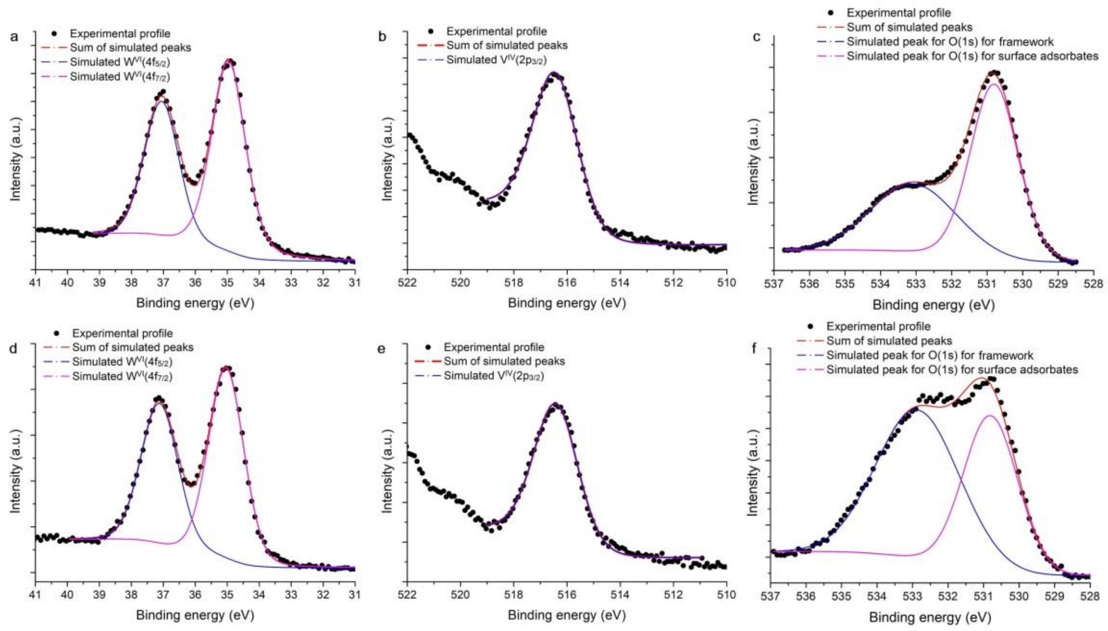

Supplementary Figure 5. XPS profiles. (**a**) W in **VT-1**, (**b**) V in **VT-1**, (**c**) O in **VT-1**, (**d**) W in **VT-5**, (**e**) V in **VT-5**, and (**f**) O in **VT-5**.

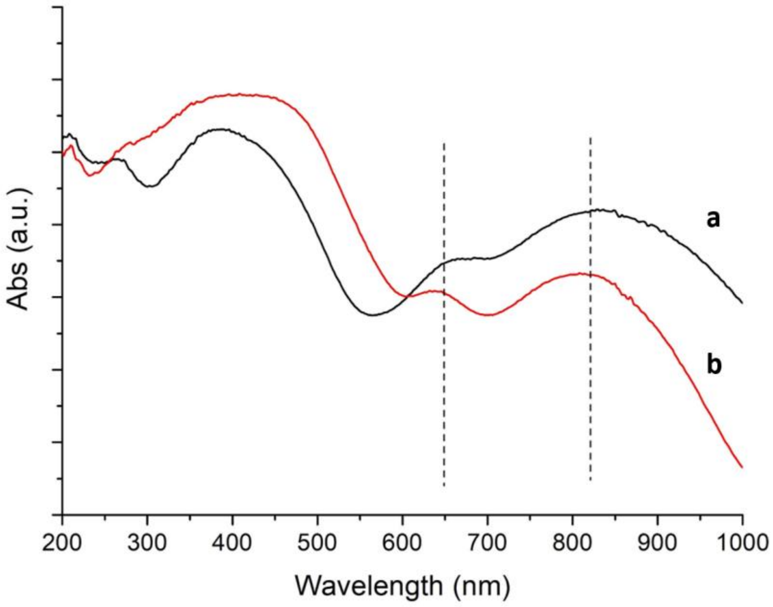

Supplementary Figure 6. DR-UV-vis spectra. (**a**) **VT-1** and (**b**) **VT-5**.

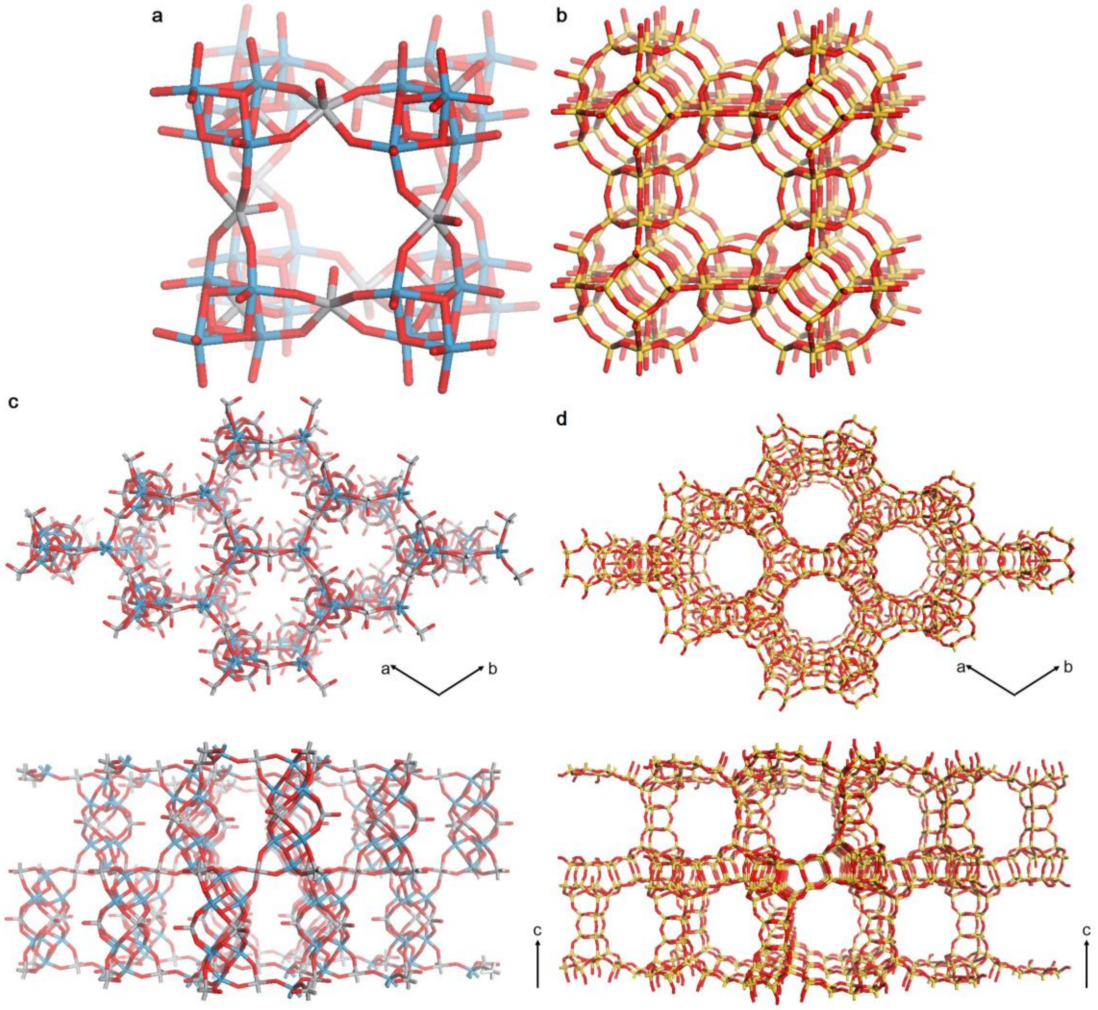

Supplementary Figure 7. Comparison of the structures of VT with those of zeolites. (**a**) **VT-1**, (**b**) LTA zeolite, (**c**) **VT-5**, and (**d**) IRY zeolite; W (blue), V (grey), O (red), and Si (yellow).

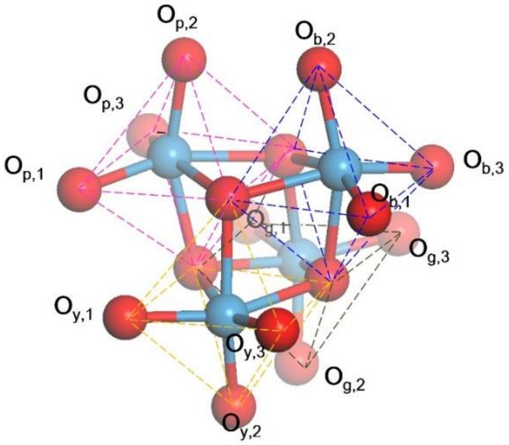

Supplementary Figure 8. Ball-and-stick representation of the cubane unit of $[W_4O_{16}]^{8-}$. W (blue), O (red).

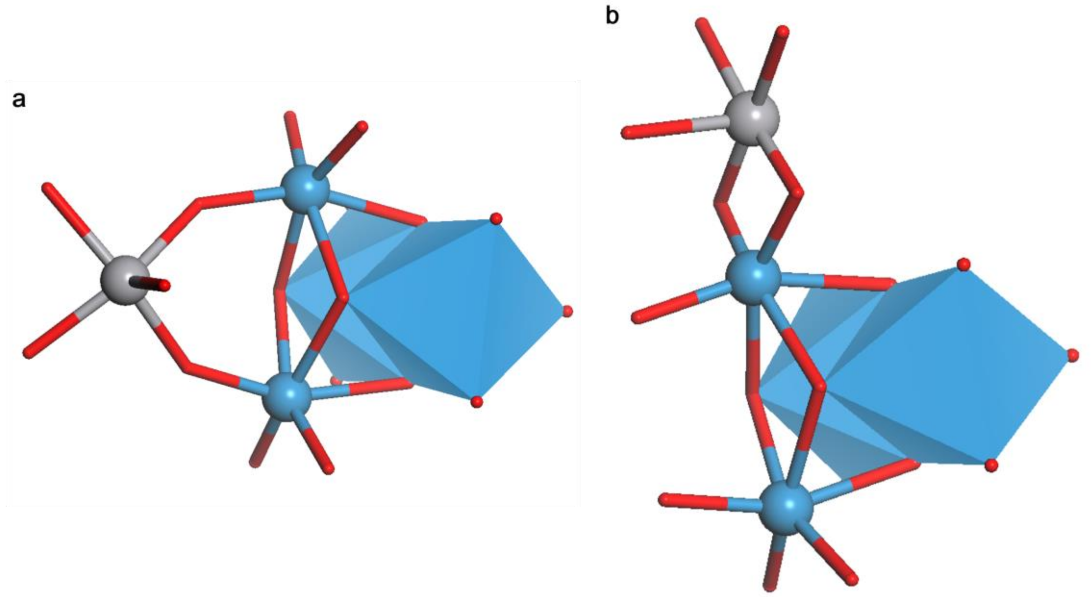

Supplementary Figure 9. Structural models of the connections. (**a**) Type i connection and (**b**) type ii connection, W (blue), V (grey), and O (red).

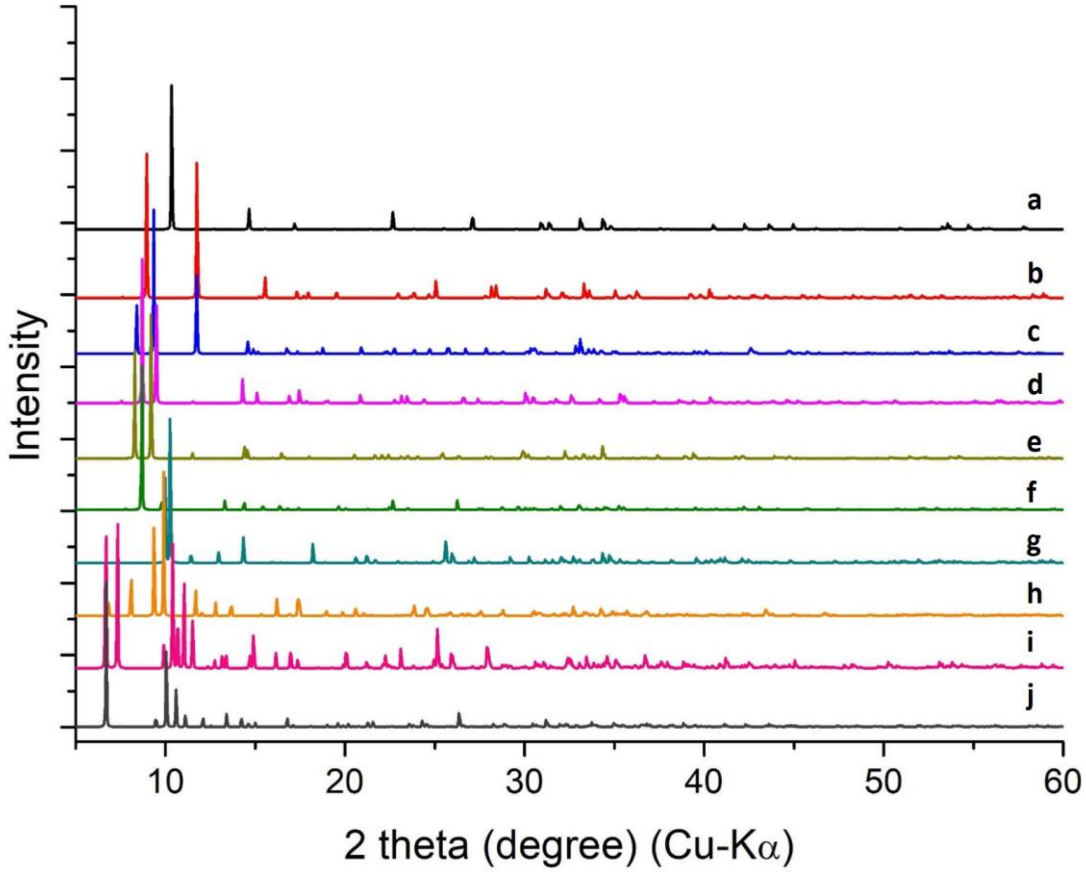

Supplementary Figure 10. Simulated XRD patterns of the VT family. (**a**) **VT-1**, (**b**) **VT-2**, (**c**) **VT-3**, (**d**) **VT-4**, (**e**) **VT-5**, (**f**) **VT-6**, (**g**) **VT-7**, (**h**) **VT-8**, (**i**) **VT-9**, and (**j**) **VT-10.**

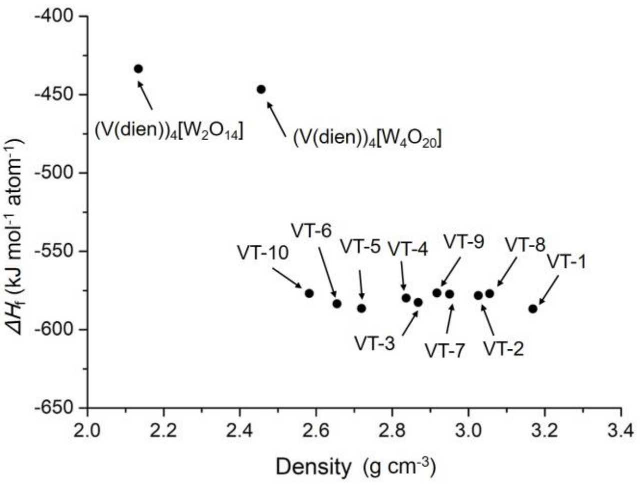

Supplementary Figure 11. Formation energy-density map for the molecular type VTs and the proposed zeolitic VT structures.

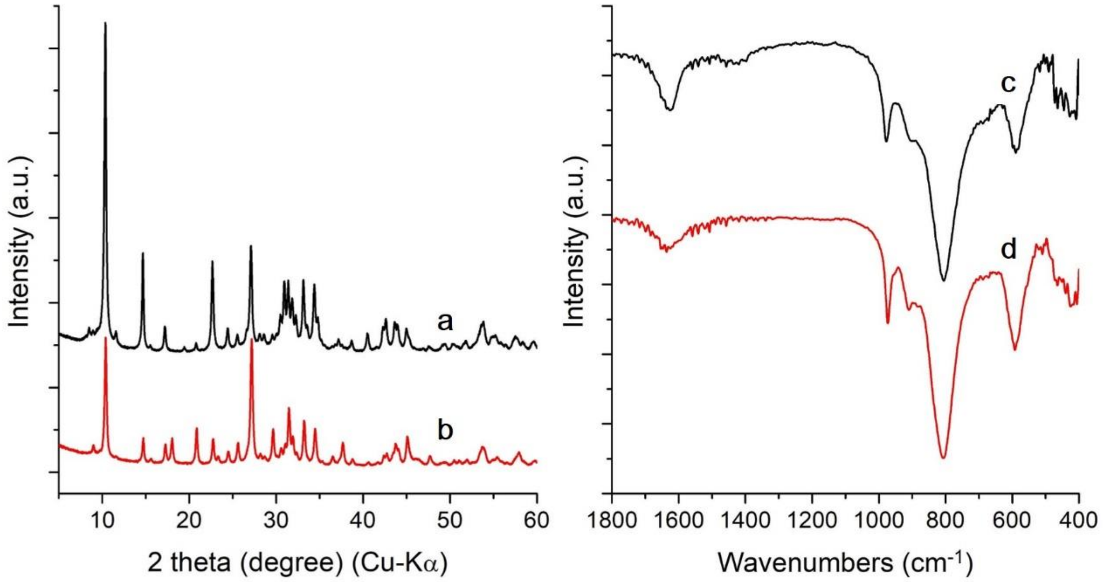

Supplementary Figure 12. XRD patterns and FTIR spectra of VT-1 before and after Cs exchange. XRD patterns of (**a**) **VT-1** and (**b**) **Cs-VT-1** and FTIR spectra of (**c**) **VT-1** and (**d**) **Cs-VT-1.**

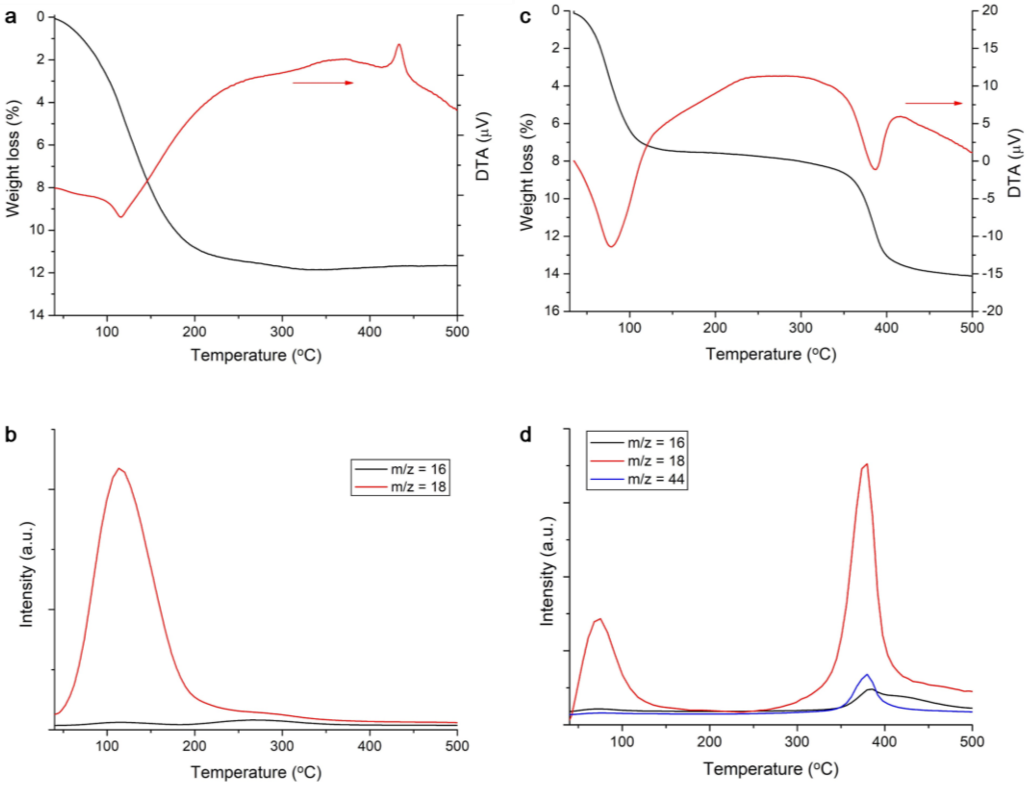

Supplementary Figure 13. Results of thermal analysis using TG-DTA and TPD-MS. (**a**) TG-DTA and (**b**) TPD-MS profiles of **VT-1;** (**c**) TG-DTA and (**d**) TPD-MS profiles of **VT-5**. The peaks at m/z = 16, m/z = 18, and m/z = 44 were ascribed to NH_3, H_2O, and CO_2, respectively.

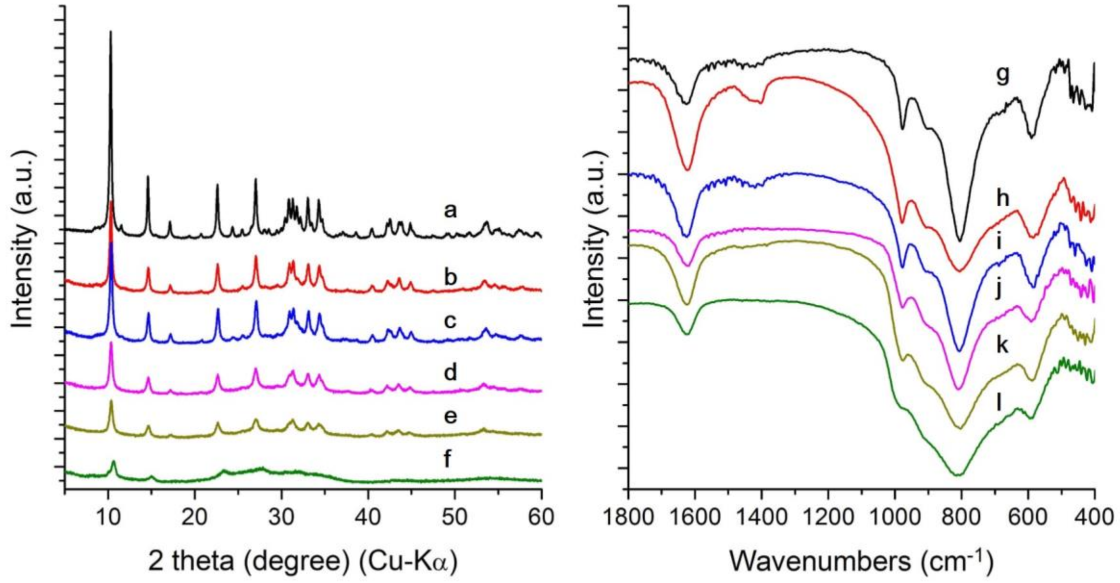

Supplementary Figure 14. XRD patterns and FTIR spectra of VT-1 calcined at different temperatures. XRD patterns of (**a**) **VT-1** and **VT-1** calcined at (**b**) 150 ºC, (**c**) 200 ºC, (**d**) 250 ºC, (**e**) 300 ºC, and (**f**) 350 ºC under N_2 and FTIR spectra of (**g**) **VT-1** and **VT-1** calcined at (**h**) 150 ºC, (**i**) 200 ºC, (**j**) 250 ºC, (**k**) 300 ºC, and (**l**) 350 ºC under N_2.

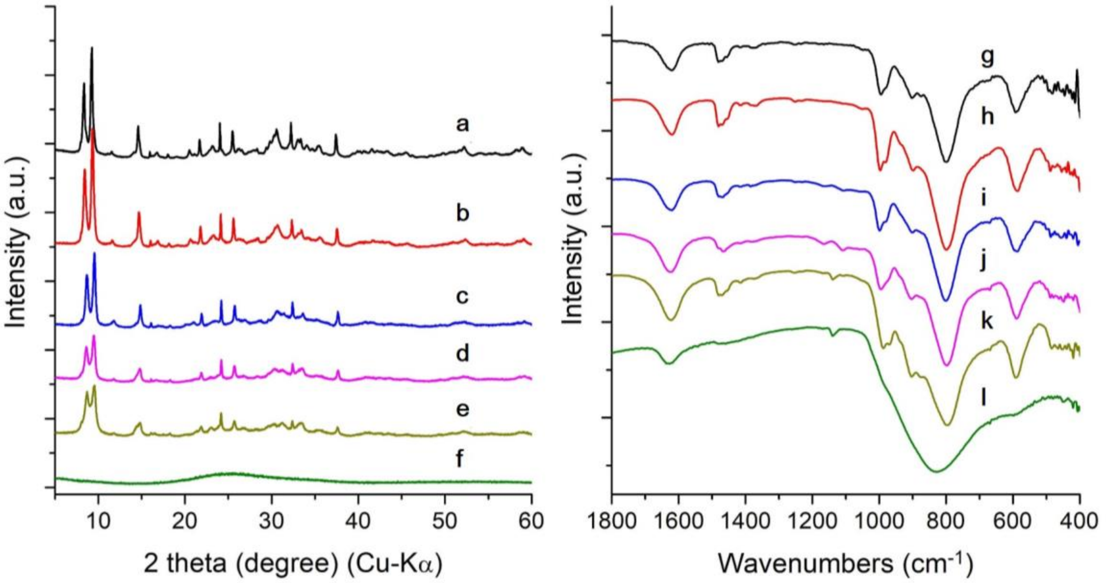

Supplementary Figure 15. XRD patterns and FTIR spectra of VT-5 calcined at different temperatures. XRD patterns of (**a**) **VT-5** and **VT-5** calcined at (**b**) 150 ºC, (**c**) 200 ºC, (**d**) 250 ºC, (**e**) 300 ºC, and (**f**) 350 ºC under N_2 and FTIR spectra of (**g**) **VT-5** and **VT-5** calcined at (**h**) 150 ºC, (**i**) 200 ºC, (**j**) 250 ºC, (**k**) 300 ºC, and (**l**) 350 ºC under N_2.

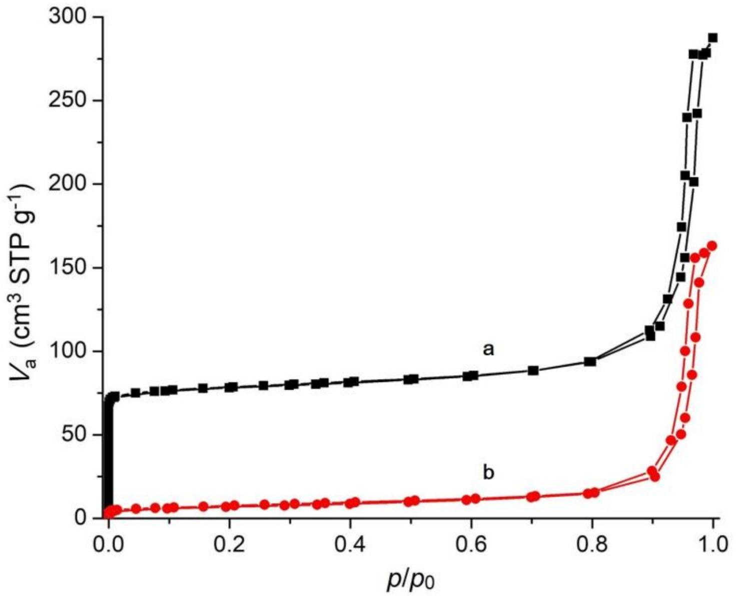

Supplementary Figure 16. N_2 adsorption-desorption isotherms. (**a**) **VT-1** and (**b**) **VT-1** calcined at 350 ^{o}C.

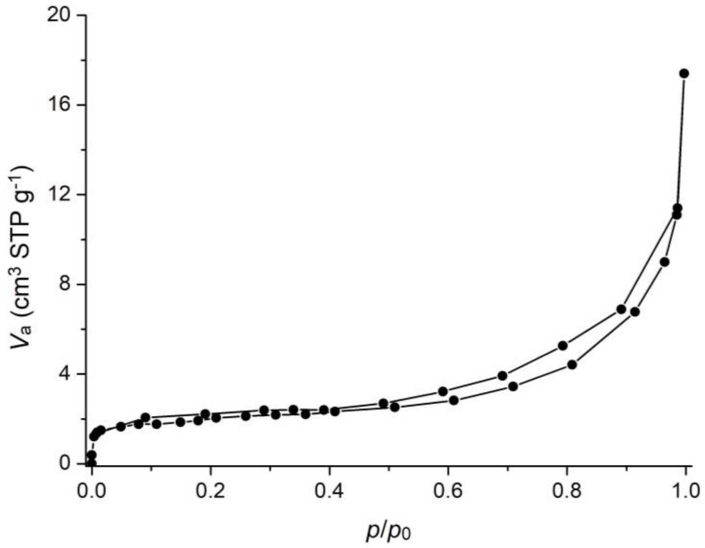

Supplementary Figure 17. N_2 adsorption-desorption isotherm of VT-5.

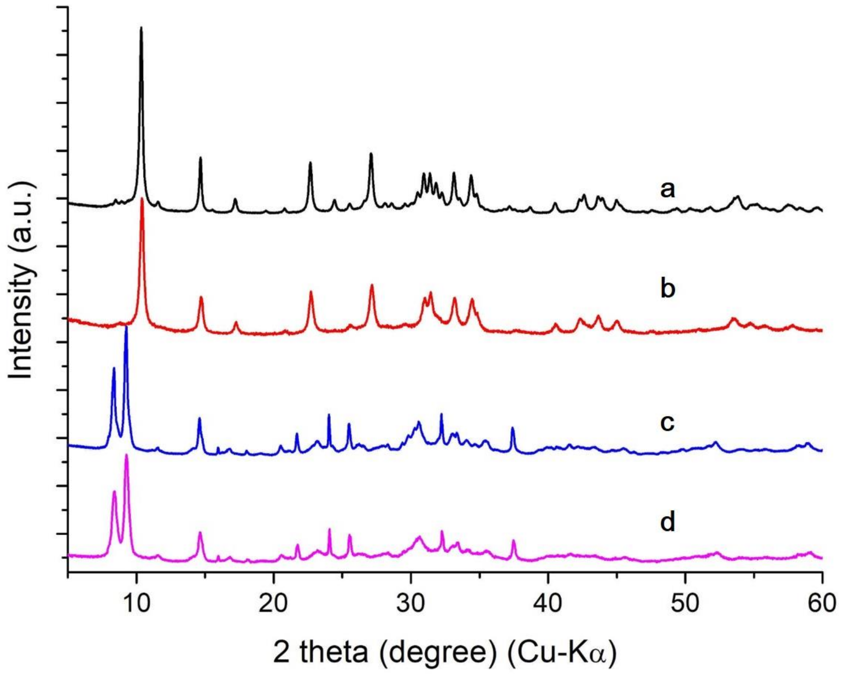

Supplementary Figure 18. XRD patterns of the materials before and after adsorption. (**a**) **VT-1**, (**b**) **VT-1** after adsorption, (**c**) **VT-5**, and (**d**) **VT-5** after adsorption.

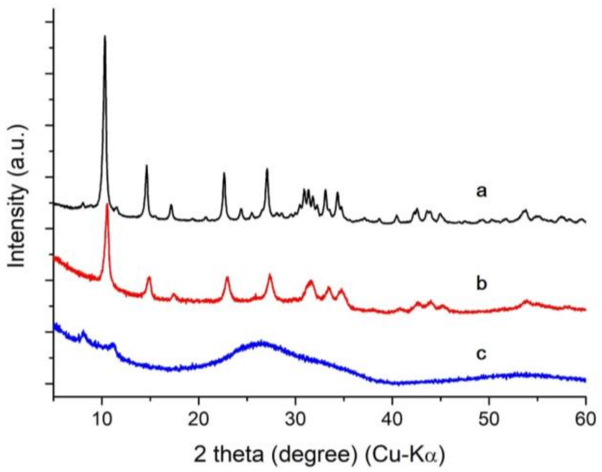

Supplementary Figure 19. XRD patterns of VT-1 before and after reaction. (**a**) **VT-1** before reaction, (**b**) **VT-1** after reaction at 120 ^oC, and (**c**) **VT-1** after reaction at 200 ^oC.

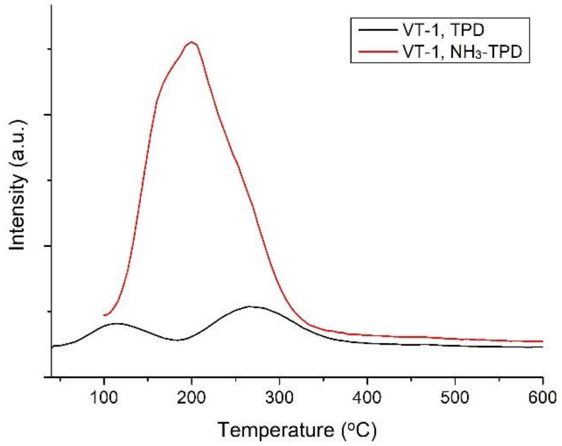

Supplementary Figure 20. TPD and NH_3-TPD profile (m/z = 16) of VT-1.

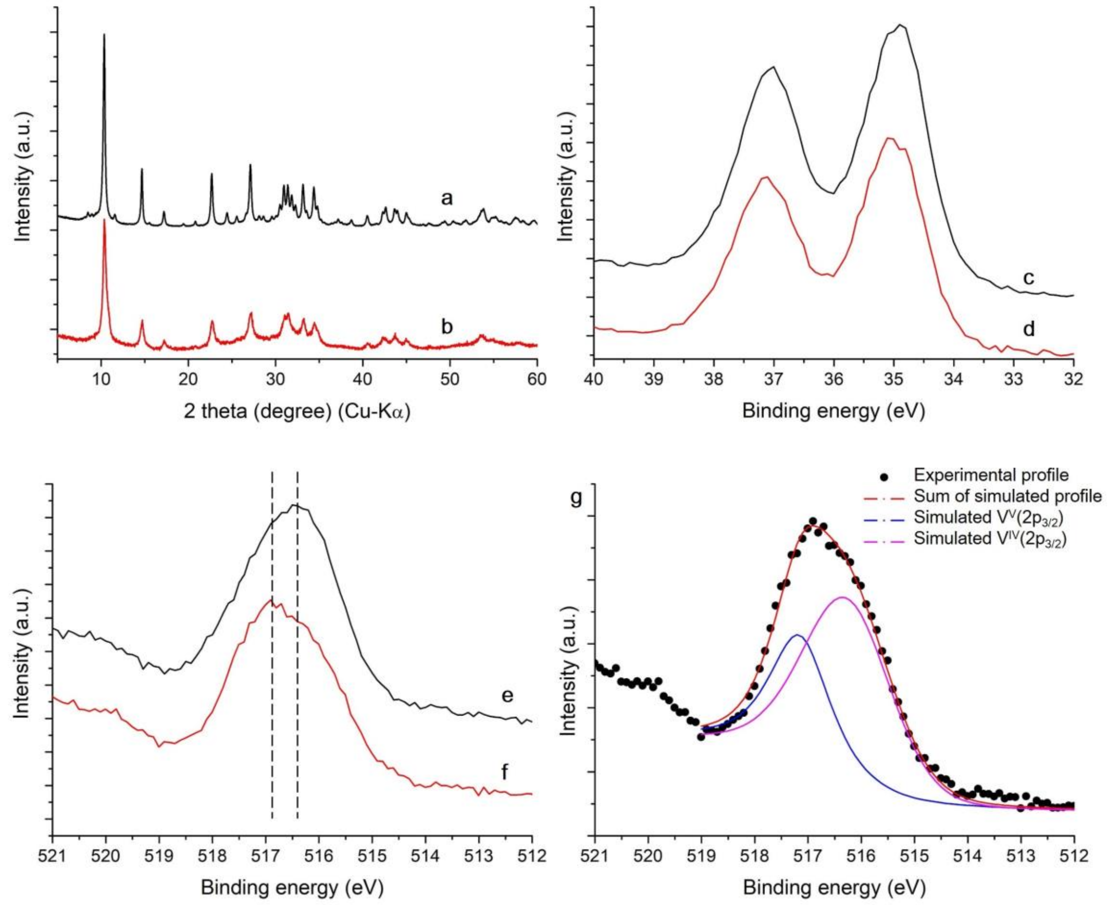

Supplementary Figure 21. Characterizations of VT-1 and VT-1 calcined at 120 °C in air. XRD patterns of (**a**) **VT-1** and (**b**) **VT-1** calcined at 120 °C in air; XPS for W of (**c**) **VT-1** and (**d**) **VT-1** calcined at 120 °C in air; XPS for V of (**e**) **VT-1** and (**f**) **VT-1** calcined at 120 °C in air; (**g**) curve fitting for V in **VT-1** calcined at 120 °C in air, $V^{V}/V^{IV} = 0.54$.

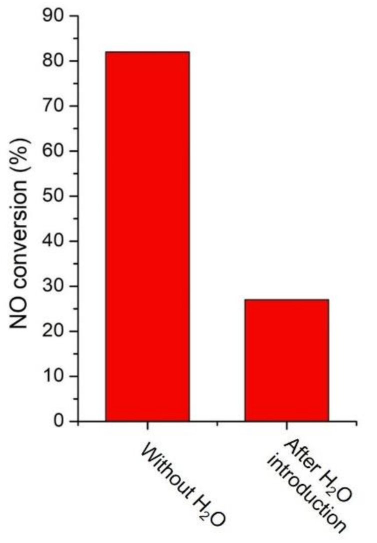

Supplementary Figure 22. Water effect for NH_3-SCR. Reaction conditions: **VT-1**: 0.15 g with the secondary particle size of 38-106 μm, total flow rate: 250 mL min^{-1}, NO: 250 ppm, NH_3: 250 ppm, O_2 4 vol% (Ar as the diluter), temperature: 150 ^oC, water 2.2 vol% when used.

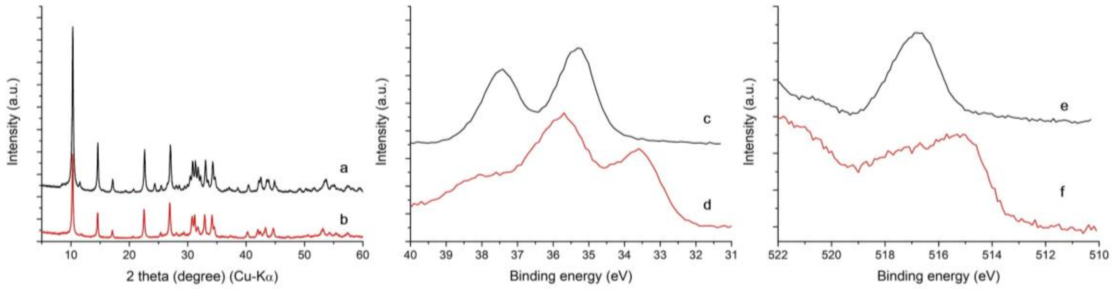

Supplementary Figure 23. Characterizations of VT-1 after SO_2 treatment. XRD patterns of **VT-1** (**a**) before and (**b**) after SO_2 treatment at 130 ^oC, XPS profile of W of **VT-1** (**c**) before and (**d**) after SO_2 treatment at 130 ^oC, XPS profile of V of **VT-1** (**e**) before and (**f**) after SO_2 treatment at 130 ^oC. SO_2 was generate by reacting Na_2SO_3 with HCl. HCl (37%) was dropped (ca. 4 drops per minute) to Na_2SO_3 (20 g) continuously to form SO_2 gas that was flowed to **VT-1** at 130 ^oC directly for 1 h.

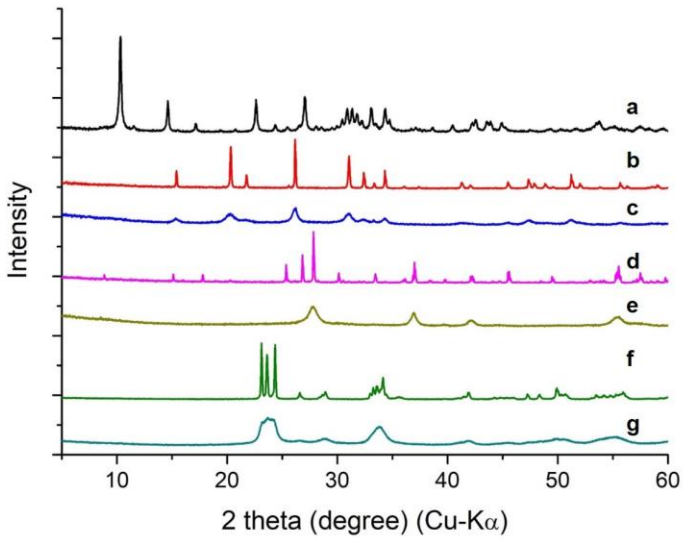

Supplementary Figure 24. XRD patterns of the materials before and after ball-milling for 100 min. (**a**) **VT-1**, (**b**) V_2O_5, (**c**) V_2O_5 after ball-milling, (**d**) VO_2, (**e**) VO_2 after ball-milling, (**f**) WO_3, and (**g**) WO_3 after ball-milling.

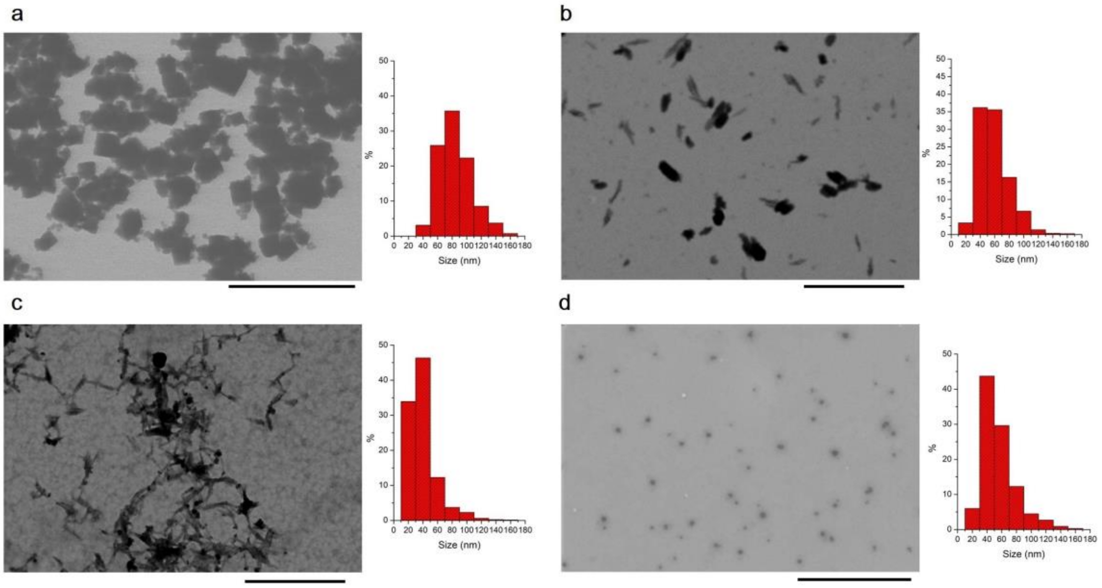

Supplementary Figure 25. TEM images (left) and corresponding size distribution (right) of the materials. (**a**) **VT-1**, scale bar: 500 nm, (**b**) V_2O_5, scale bar: 500 nm, (**c**) VO_2, scale bar: 500 nm, and (**d**) WO_3 scale bar: 2 μm.

Supplementary Tables

Supplementary Table 1. Synthetic conditions for **VT-1** using different V sources. [a]

Entry	W source	Base	V source	pH	*T* (°C)	Time (h)	Solid [b]
1	WO_3	KOH	$VOSO_4$	4.0	175	8	Y
2	WO_3	KOH	-	4.0	175	8	N
3	WO_3	KOH	NH_4VO_3	4.0	175	8	N
4	WO_3	KOH	$NaVO_3$	4.0	175	8	N
5	WO_3	KOH	V_2O_5	4.0	175	8	Y
6	WO_3	KOH	VO_2	4.0	175	8	Y
7	WO_3	KOH	V_2O_3	4.0	175	8	Y

[a] W: 20 mmol based on, KOH: 65 mmol, V source: 11 mmol based on V, water: 200 mL, H_2SO_4 (1M) was used for pH adjustment; [b] solid obtained: Y, solid not obtained: N.

Supplementary Table 2. Crystallographic information and Rietveld refinement parameters for **VT-1**, **VT-5**, and **Cs-VT-1** obtained from powder XRD.

	**VT-1**	**VT-5**	**Cs-VT-1**
crystal system	Cubic	Trigonal	Cubic
space group	PA-3	P321	PA-3
a (Å)	17.1101	11.77849	17.0497
b (Å)	17.1101	11.77849	17.0497
c (Å)	17.1101	22.08607	17.0497
agreement factors			
R_{wp}	6.17%	6.36%	7.44%
$R_{wp(w/o\ bck)}$	12.71%	16.80%	15.90%
R_p	4.66%	4.76%	5.79%
pattern parameter			
peak shape function	Pseudo-Voigt	Pseudo-Voigt	Pseudo-Voigt
FWHM	U = 0.63257,<br>V = -0.28658,<br>W = 0.09655	U = 0.03787,<br>V = -0.01444,<br>W = 0.00138	U = 0.72478,<br>V = -0.29898,<br>W = 0.10043
profile parameter	N_A = 1.05713,<br>N_B = -0.01163	N_A = 0.69280,<br>N_B = -0.00223	N_A = 0.90131,<br>N_B = -0.00332
line shift			
instrument geometry	Bragg-Brentano	Bragg-Brentano	Bragg-Brentano
zero point	-0.36975	-0.03039	0.45483
shift#1	0.33707	0.03693	-0.38898
shift#2	0.06213	-0.01985	-0.12436
correction:			
method	Berar-Baldinozzi	Berar-Baldinozzi	Berar-Baldinozzi
parameter	$P1$ = 0.02340,<br>$P2$ = -0.11328,<br>$P3$ = -0.05876,<br>$P4$ = 0.22166	$P1$ = 0.01000,<br>$P2$ = 0.00000,<br>$P3$ = 0.00000,<br>$P4$ = 0.00000	$P1$ = -0.12431,<br>$P2$ = 0.04255,<br>$P3$ = 0.16650,<br>$P4$ = -0.08754
background coefficients	polynomial = 100	polynomial = 100	polynomial = 20
preferred orientation function	March-Dollase	March-Dollase	March-Dollase
	$R0$ = 1.0	$R0$ = 0.75977	$R0$ = 1.0
Crystallite Size	-	A = 26197.15318<br>B = 26197.15318<br>C = 1500.49102	-
Lattice Strain	-	A = 2.04014<br>B = 2.04014<br>C = 0.11385	-

Supplementary Table 3. Results of the charge-flipping algorithm for **VT-1** and the corresponding structure.

Peaks	*X*	*Y*	*Z*	Intensity	Assignment
Q1	0.1614	0.6966	0.3267	40.64	W
Q2	0.1964	0.3037	0.6961	33.22	W
Q3	0.0958	0.5958	0.904	11.55	Cation or water
Q4	0.0069	0.2472	0.2716	7.97	V
Q5	0.1735	0.7249	0.217	9.83	O
Q6	0.1482	0.3518	0.649	9.7	Cation or water
Q7	0.0928	0.6893	0.694	7.19	O
Q8	0.0669	0.702	0.3083	5.99	O
Q9	0.1969	0.6969	0.803	7.14	O
Q10	0.0292	0.4525	0.9271	5.88	Cation or water

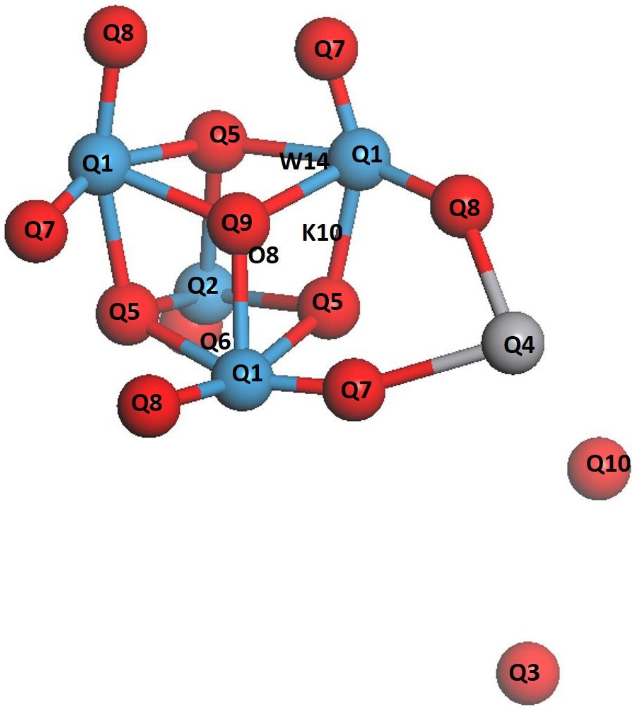

Supplementary Table 4. Atomic position of **VT-1** after Rietveld refinement and the corresponding structure.

Atom	*X*	*Y*	*Z*	U_{iso}	Occupancy
W1	0.16135	0.69603	0.33109	1E-3	1
V2	0.01212	0.25738	0.7908	0.02	1
O3	0.82643	0.05336	0.80402	0	1
O4	0.69244	0.06037	0.66033	0	1
O5	0.83254	0.16277	0.90485	0	1
O6	0.19817	0.30645	0.59259	0	1
O7	0.21209	0.29795	0.84008	0	1
O8	0.3309	0.52117	0.67583	0	1
O9	0.04917	0.43208	0.91757	0	1
O10	0.41981	0.42572	0.36977	0	1
O11	0.72059	0.56388	0.48593	0	1
O12	0.36596	0.5171	0.52892	0	0.74
O13	0.98405	0.63519	0.49299	0	1
O14	0.67858	0.44178	0.62341	0	1
O15	0.63219	0.40967	0.59365	0	0.98
W16	0.19218	0.30782	0.69218	1E-3	1
O17	0.33333	0.33333	0.33333	0.06	1

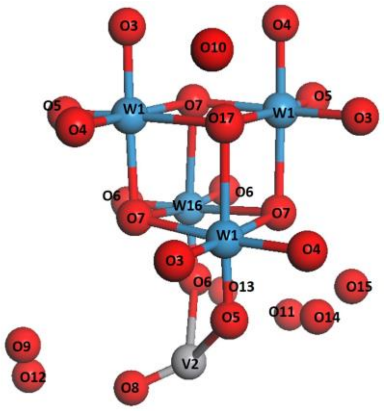

Supplementary Table 5. BVS of **VT-1** and the corresponding structure.

	Bond length	BVS
W-O	1.836	
	2.087	
	2.117	
	2.097	
	1.831	
	1.836	5.641
V-O	1.586	
	1.929	
	1.916	
	1.905	
	1.912	3.952

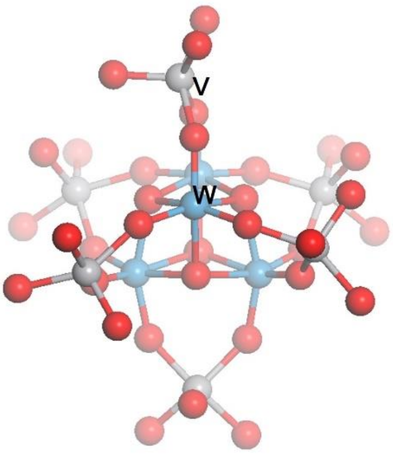

Supplementary Table 6. Comparison of inorganic crystalline microporous materials.

	Zeolite	Tetrahedral-octahedral framework	Zeolitic transition metal oxide
Composition	Main group elementals	Main group elementals, transition metal elements	Fully-transition metal elements
Central elements coordination	4	4,6	4,5,6 or more
Position of transition metal	-	Framework, pore	Framework
Roll of transition metal	-	Framework construction, pore occupation	Framework construction
Typical example	4 A, ZSM-5	Tschörtnerite [1]<br>Pharmacosiderite [2]<br>ETS [3]	Zeolitic **VT**s (the present work)

Supplementary Table 7. All the possible connections of $[W_4O_{16}]^{8-}$ with metal ion linkers

Entry	The number of type i and type ii connection	Combination of octahedra for the type i connection	The rest octahedra for the type ii connection	Reasonable combination of octahedra for the cluster
1	6 type i, 0 type ii	(b, g), (b, p), (b, y), (g, p), (g, y), (p, y)	-	type i: (b, g), (b, p), (b, y), (g, p), (g, y), (p, y)
2	5 type i, 1 type ii	(b, p), (b, y), (g, p), (g, y), (p, y)	b, g	-
3	5 type i, 1 type ii	(b, g), (b, y), (g, p), (g, y), (p, y)	b, p	-
4	5 type i, 1 type ii	(b, g), (b, p), (g, p), (g, y), (p, y)	b, y	-
5	5 type i, 1 type ii	(b, g), (b, p), (b, y), (g, y), (p, y)	g, p	-
6	5 type i, 1 type ii	(b, g), (b, p), (b, y), (g, p), (p, y)	g, y	-
7	5 type i, 1 type ii	(b, g), (b, p), (b, y), (g, p), (g, y)	p, y	-
8	4 type i, 2 type ii	(b, y), (g, p), (g, y), (p, y)	b, g, b, p	-
9	4 type i, 2 type ii	(b, p), (g, p), (g, y), (p, y)	b, g, b, y	-
10	4 type i, 2 type ii	(b, p), (b, y), (g, y), (p, y)	b, g, g, p	-
11	4 type i, 2 type ii	(b, p), (b, y), (g, p), (p, y)	b, g, g, y	-
12	4 type i, 2 type ii	(b, p), (b, y), (g, p), (g, y)	b, g, p, y	-
13	4 type i, 2 type ii	(b, g), (g, p), (g, y), (p, y)	b, p, b, y	-
14	4 type i, 2 type ii	(b, g), (b, y), (g, y), (p, y)	b, p, g, p	-
15	4 type i, 2 type ii	(b, g), (b, y), (g, p), (p, y)	b, p, g, y	-
16	4 type i, 2 type ii	(b, g), (b, y), (g, p), (g, y)	b, p, p, y	-
17	4 type i, 2 type ii	(b, g), (b, p), (g, y), (p, y)	b, y, g, p	-
18	4 type i, 2 type ii	(b, g), (b, p), (g, p), (p, y)	b, y, g, y	-
19	4 type i, 2 type ii	(b, g), (b, p), (g, p), (g, y)	b, y, p, y	-
20	4 type i, 2 type ii	(b, g), (b, p), (b, y), (p, y)	g, p, g, y	-
21	4 type i, 2 type ii	(b, g), (b, p), (b, y), (g, y)	g, p, p, y	-
22	4 type i, 2 type ii	(b, g), (b, p), (b, y), (g, p)	g, y, p, y	-
23	3 type i, 3 type ii	(b, g), (b, p), (b, y)	g, p, g, y, p, y	type i: (b, g), (b, p), (b, y), type ii: (g, g), (p, p), (y, y)
24	3 type i, 3 type ii	(b, g), (b, p), (g, p)	b, y, g, y, p, y	-
25	3 type i, 3 type ii	(b, g), (b, p), (g, y)	b, y, g, p, p, y	-
26	3 type i, 3 type ii	(b, g), (b, p), (p, y)	b, y, g, p, g, y	-
27	3 type i, 3 type ii	(b, g), (b, y), (g, p)	b, p, g, y, p, y	-
28	3 type i, 3 type ii	(b, g), (b, y), (g, y)	b, p, g, p, p, y	-
29	3 type i, 3 type ii	(b, g), (b, y), (p, y)	b, p, g, p, g, y	-
30	3 type i, 3 type ii	(b, g), (g, p), (g, y)	b, p, b, y, p, y	type i: (b, g), (g, p), (g, y), type ii: (b, b), (p, p), (y, y)
31	3 type i, 3 type ii	(b, g), (g, p), (p, y)	b, p, b, y, g, y	-
32	3 type i, 3 type ii	(b, g), (g, y), (p, y)	b, p, b, y, g, p	-
33	3 type i, 3 type ii	(b, p), (b, y), (g, p)	b, g, g, y, p, y	-
34	3 type i, 3 type ii	(b, p), (b, y), (g, y)	b, g, g, p, p, y	-
35	3 type i, 3 type ii	(b, p), (b, y), (p, y)	b, g, g, p, g, y	-
36	3 type i, 3 type ii	(b, p), (g, p), (g, y)	b, g, b, y, p, y	-
37	3 type i, 3 type ii	(b, p), (g, p), (p, y)	b, g, b, y, g, y	type i: (b, p), (g, p), (p, y), type ii: (b, b), (g, g), (y, y)
38	3 type i, 3 type ii	(b, p), (g, y), (p, y)	b, g, b, y, g, p	-
39	3 type i, 3 type ii	(b, y), (g, p), (g, y)	b, g, b, p, p, y	-
40	3 type i, 3 type ii	(b, y), (g, p), (p, y)	b, g, b, p, g, y	-
41	3 type i, 3 type ii	(b, y), (g, y), (p, y)	b, g, b, p, g, p	type i: (b, y), (g, y), (p, y), type ii: (b, b), (g, g), (p, p)
42	3 type i, 3 type ii	(g, p), (g, y), (p, y)	b, g, b, p, b, y	-
43	2 type i, 4 type ii	(b, g), (b, p)	b, y, g, p, g, y, p, y	-
44	2 type i, 4 type ii	(b, g), (b, y)	b, p, g, p, g, y, p, y	-
45	2 type i, 4 type ii	(b, g), (g, p)	b, p, b, y, g, y, p, y	-
46	2 type i, 4 type ii	(b, g), (g, y)	b, p, b, y, g, p, p, y	-
47	2 type i, 4 type ii	(b, g), (p, y)	b, p, b, y, g, p, g, y	type i: (b, g), (p, y), type ii: (b, b), (g, g), (p, p), (y, y)
48	2 type i, 4 type ii	(b, p), (b, y)	b, g, g, p, g, y, p, y	-
49	2 type i, 4 type ii	(b, p), (g, p)	b, g, b, y, g, y, p, y	-
50	2 type i, 4 type ii	(b, p), (g, y)	b, g, b, y, g, p, p, y	type i: (b, p), (g, y), type ii: (b, b), (g, g), (p, p), (y, y)
51	2 type i, 4 type ii	(b, p), (p, y)	b, g, b, y, g, p, g, y	-
52	2 type i, 4 type ii	(b, y), (g, p)	b, g, b, p, g, y, p, y	type i: (b, y), (g, p), type ii: (b, b), (g, g), (p, p), (y, y)
53	2 type i, 4 type ii	(b, y), (g, y)	b, g, b, p, g, p, p, y	-
54	2 type i, 4 type ii	(b, y), (p, y)	b, g, b, p, g, p, g, y	-
55	2 type i, 4 type ii	(g, p), (g, y)	b, g, b, p, b, y, p, y	-
56	2 type i, 4 type ii	(g, p), (p, y)	b, g, b, p, b, y, g, y	-
57	2 type i, 4 type ii	(g, y), (p, y)	b, g, b, p, b, y, g, p	-
58	1 type i, 5 type ii	(b, g)	b, p, b, y, g, p, g, y, p, y	-
59	1 type i, 5 type ii	(b, p)	b, g, b, y, g, p, g, y, p, y	-
60	1 type i, 5 type ii	(b, y)	b, g, b, p, g, p, g, y, p, y	-
61	1 type i, 5 type ii	(g, p)	b, g, b, p, b, y, g, y, p, y	-
62	1 type i, 5 type ii	(g, y)	b, g, b, p, b, y, g, p, p, y	-
63	1 type i, 5 type ii	(p, y)	b, g, b, p, b, y, g, p, g, y	-
64	0 type i, 6 type ii		b, g, b, p, b, y, g, p, g, y, p, y	-

Supplementary Table 8. Lattice parameters of the **VT** family after geometry optimization.

	**VT-1**	**VT-2**	**VT-3**	**VT-4**	**VT-5**	**VT-6**	**VT-7**	**VT-8**	**VT-9**	**VT-10**
a (Å)	17.09707	11.4012	12.1432	11.7296	12.3065	12.3137	17.7096	13.2618	26.4717	26.4052
b (Å)	17.09707	11.4012	12.1432	11.7296	12.3065	12.3137	11.0990	8.9338	8.5027	26.4052
c (Å)	17.09707	11.6250	21.6235	23.4325	22.2021	34.0734	13.6551	11.2116	24.1200	8.7970
α (Å)	90	90	90	90	90	90	90	90.1162	90	90
β (Å)	90	90	90	90	90	90	90	77.3001	90	90
γ (Å)	90	120	120	120	120	120	90	90.2942	90	90
Space group	PA-3	P3	P3	P321	P321	R3	AMA2	P1	PNMA	P4MM

Supplementary Table 9. Atomic positions of **VT-1** after geometry optimization.

Atom	*X*	*Y*	*Z*
W1	0.17965	0.68322	0.3188
V2	5.7E-4	0.27156	0.77415
O3	0.80363	0.07455	0.80092
O4	0.69198	0.07567	0.69561
O5	0.8002	0.19008	0.92204
O6	0.19127	0.29782	0.5777
O7	0.2001	0.30267	0.80326
O8	0.33888	0.50312	0.66207
W10	0.18296	0.31704	0.68296
O11	0.30379	0.30379	0.30379
W1	0.17965	0.68322	0.3188

Supplementary Table 10. Atomic positions of **VT-2** after geometry optimization.

Atom	*X*	*Y*	*Z*
O1	0.29729	0.38468	0.52125
O2	0.59156	0.53498	0.29501
O3	0.52534	0.59421	0.52076
O4	0.38787	0.30477	0.31708
O5	0.35975	0.53875	0.3258
O6	0.58943	0.02934	0.08541
O7	0.63309	0.17812	0.9032
W8	0.47889	0.85569	0.62686
V9	0.51951	0.95883	0.3937
V10	0.4011	0.40121	0.91539
O11	0.36929	0.54644	-0.07136
O12	0.77268	0.29199	0.10678
O13	0.48288	0.71233	0.72579
O14	0.025	0.57757	0.74613
W15	0.62466	0.14573	0.2052
O16	0.26126	0.26286	0.9114
W17	0.33333	0.66667	0.85364
O18	0.33333	0.66667	0.55351
W19	0.66667	0.33333	-0.02213
O20	0.66667	0.33333	0.28243

Supplementary Table 11. Atomic positions of **VT-3** after geometry optimization.

Atom	*X*	*Y*	*Z*
O1	0.90649	0.30237	0.03176
O2	-0.00265	0.22952	0.43341
O3	0.12316	0.70553	0.52653
O4	1.00787	0.7731	0.9327
O5	0.69142	0.10806	0.03153
O6	0.76592	0.98212	0.43578
O7	0.32822	0.91181	0.52381
O8	0.23517	0.02229	0.93378
O9	0.80983	0.40366	0.13721
O10	0.19	0.60487	0.63328
W11	0.74625	0.51917	0.08468
V12	0.87541	0.71397	-0.00769
O13	0.93114	0.8426	0.22404
O14	0.86382	0.69867	0.32364
W15	0.91376	0.81785	0.38545
W16	0.21868	0.48336	0.58076
V17	0.16833	0.35269	0.46301
O18	0.06524	0.15744	0.7221
O19	0.13161	0.30212	0.82211
W20	0.08177	0.18198	0.88294
V21	0.78748	0.12205	0.23492
O22	0.07532	0.93565	0.33079
V23	0.22196	0.88763	0.73313
O24	0.92255	0.06234	0.82891
O25	0.36758	0.18374	0.14844
O26	0.63258	0.78919	0.64372
O27	0.73743	0.24905	0.24593
O28	0.26897	0.75742	0.74174
O29	0.93391	0.82604	0.04102
O30	0.22162	0.45929	0.41149
O31	0.33665	0.34387	0.23147
O32	-0.01825	0.33419	0.73497
W33	0.66667	0.33333	0.20741
O34	0.66667	0.33333	0.0428
W35	0.33333	0.66667	0.70391
O36	0.33333	0.66667	0.5395
W37	0	0	0.26046
O38	0	0	0.42652
W39	0	0	0.75815
O40	0	0	0.92373

Supplementary Table 12. Atomic positions of **VT-4** after geometry optimization.

Atom	*X*	*Y*	*Z*
O1	0.34639	0.42237	0.69201
O2	0.65361	0.57763	0.19201
O3	0.64476	0.55282	0.30873
O4	0.35524	0.44718	0.80873
W5	0.44326	0.85602	0.63923
W6	0.55674	0.14398	0.13923
O7	0.401	0.57682	0.49037
O8	0.26292	0.74472	0.59088
O9	0.48378	0.50828	0.58159
O10	0.599	0.42318	0.99037
O11	0.73708	0.25528	0.09088
O12	0.51622	0.49172	0.08159
V13	0.03029	0.51514	0.75
O14	0.87454	0.43727	0.75
V15	0.46296	0	0.5
V16	0.53704	0	0
O17	1	0.32756	0.5
O18	0.32756	0.32756	0
W19	0.33333	0.66667	0.52668
O20	0.33333	0.66667	0.67715
W21	0.33333	0.66667	0.97332
O22	0.33333	0.66667	0.82285

Supplementary Table 13. Atomic positions of **VT-5** after geometry optimization.

Atom	*X*	*Y*	*Z*
O1	0.30316	0.43091	0.68978
O2	0.64942	0.54641	0.19086
O3	0.64864	0.54419	0.30926
O4	0.30409	0.43261	0.80881
W5	0.43741	0.84506	0.64063
W6	0.59105	0.15486	0.14137
O7	0.40048	0.57664	0.48587
O8	0.27552	0.74621	0.58777
O9	0.46257	0.49458	0.58256
O10	0.57211	0.39665	0.98712
O11	0.74623	0.27566	0.08842
O12	0.4922	0.4607	0.08406
V13	0.2195	0.8239	0.74979
O14	0.15806	0.91033	0.7497
V15	0.48024	0	0.5
V16	0.55585	0	0
O17	0	0.3515	0.5
O18	0.31542	0.31542	0
W19	0.33333	0.66667	0.51954
O20	0.33333	0.66667	0.68089
W21	0.33333	0.66667	0.97995
O22	0.33333	0.66667	0.81824

Supplementary Table 14. Atomic positions of **VT-6** after geometry optimization.

Atom	*X*	*Y*	*Z*
O1	0.56629	0.87965	0.78737
O2	0.32254	0.87713	0.86819
W3	0.15392	0.58622	0.75538
O4	0.40699	0.82375	0.65432
O5	0.26005	0.52868	0.72144
O6	0.48866	0.96809	0.71763
V8	0.6101	0.79149	0.82526
O10	0.10701	0.68697	0.86568
O11	0.34122	0.89038	0.78885
W12	0.51381	0.77049	0.90325
O13	0.40356	0.59619	0.93707
O14	0.18139	0.81494	0.94162
O15	0.14692	0.90467	0.824
O30	0.58268	0.17783	0.67126
V36	-4.6E-4	0.46121	0.66293
O37	0.66724	0.6673	0.66336
W146	0.33333	0.66667	0.67635
O147	0.33333	0.66667	0.78187
W148	0.33333	0.66667	0.98183
O149	0.33333	0.66667	0.87744

Supplementary Table 15. Atomic positions of **VT-7** after geometry optimization.

Atom	*X*	*Y*	*Z*
O1	0.4458	-0.08938	0.22646
O2	0.43963	0.08466	0.66598
O3	-0.05989	0.26791	0.33731
O4	-0.05389	-0.26615	0.55819
O5	0.56024	-0.25887	0.20188
V6	0.02882	-0.25398	0.19925
W7	-0.06929	-0.10487	0.52953
W8	-0.06976	0.10414	0.36217
O9	-0.17042	0.07725	0.37592
O10	0.44896	-0.08474	0.51055
O11	-0.17013	-0.09413	0.50694
O12	0.45076	0.08615	0.38117
O13	0.25	0.13601	0.33129
V14	-0.25	-0.0383	0.41595

Supplementary Table 16. Atomic positions of **VT-8** after geometry optimization.

Atom	*X*	*Y*	*Z*
O1	0.66405	0.62617	0.11205
O2	0.93584	0.35387	0.62299
O3	0.79646	0.35058	0.85568
O4	0.61635	0.83274	0.89863
O5	0.71776	0.15546	0.66729
W6	0.67091	0.31034	0.77185
O7	0.5427	0.63346	0.73817
O8	0.35089	0.62249	0.88587
O9	0.06592	0.35669	0.37051
O10	0.21712	0.34653	0.13517
O11	0.3941	0.83569	0.09946
O12	0.28775	0.15643	0.32788
W13	0.33905	0.31061	0.22323
O14	0.46838	0.63756	0.2585
O15	0.00712	0.73115	1.00065
O16	0.66551	0.33807	0.11979
O17	0.9369	0.63204	0.60739
O18	0.79609	0.64536	0.85497
O19	0.63254	0.15581	0.88958
O20	0.71713	0.82874	0.6812
W21	0.66129	0.67756	0.78903
O22	0.54398	0.34443	0.73856
O23	0.34595	0.33633	0.87796
O24	0.05361	0.63589	0.37884
O25	0.21535	0.64309	0.13834
O26	0.38368	0.15636	0.10852
O27	0.29069	0.82621	0.3158
W28	0.35049	0.67822	0.20644
O29	0.46282	0.34918	0.26454
O30	0.3855	0.49106	0.64925
O31	0.62372	0.49415	0.35125
O32	0.86917	0.46312	0.42663
O33	0.74005	0.49473	0.68616
O34	0.62685	0.49312	0.90867
W35	0.8893	0.51026	0.7398
W36	0.73436	0.47769	0.01353
O37	0.86438	0.491	0.04553
O38	0.98899	0.51745	0.82773
V39	0.578	0.48968	0.23241
O40	0.26482	0.49535	0.31052

O41	0.38446	0.49402	0.0902
W42	0.1199	0.51303	0.25684
W43	0.27513	0.47471	0.98446
O44	0.14918	0.49253	0.94403
O45	0.02832	0.50134	0.15791
V46	0.43226	0.48765	0.76751
V47	-0.03046	0.46868	0.48116
V48	0.00676	0.55365	-0.00424
O49	0.53503	-0.00314	0.73642
O50	0.47065	-0.00259	0.27033
V51	0.64084	-0.00621	0.77944
V52	0.36731	-0.00538	0.22195

Supplementary Table 17. Atomic positions of **VT-9** after geometry optimization.

Atom	*X*	*Y*	*Z*
O1	-1.35982	1.23215	-1.21122
O2	-1.38967	1.5764	-1.43065
O3	-1.37113	1.53378	-1.32502
O4	-1.29561	1.03836	-1.28292
O5	-1.30628	1.72289	-1.38732
W6	-1.28977	1.56616	-1.33701
O7	-1.22456	1.22367	-1.34857
O8	-1.11847	0.87746	-1.17635
V9	-1.17755	0.87938	-1.16753
O10	-1.19069	1.38262	-1.11052
O11	-1.20202	1.38134	-1.21773
W12	-1.11187	1.38117	-1.10656
W13	-1.12012	1.38982	-1.23927
O14	-1.0517	1.37437	-1.23727
O15	-1.04699	1.35392	-1.12736
O16	-1.36433	1.52917	-1.20511
O17	-1.38456	1.29727	-1.46372
O18	-1.37125	1.23322	-1.32896
O19	-1.29465	1.72093	-1.28315
O20	-1.30972	1.04146	-1.38746
W21	-1.29184	1.19397	-1.3362
O22	-1.22275	1.5315	-1.34774
O23	-1.13926	1.36201	-1.39842
V24	-1.16978	1.37194	-1.34207
O25	-1.19133	1.5	-1
O26	-1.5	1.11339	-1.30958
V27	-1.5	1.29963	-1.31388
V28	-1.36844	1.5	-1.5

Supplementary Table 18. Atomic positions of **VT-10** after geometry optimization.

Atom	*X*	*Y*	*Z*
O1	0.6458	0.72339	0.35587
O2	0.61881	0.94489	0.63725
O3	0.63349	0.83214	0.64572
O4	0.70815	0.79426	0.15737
O5	0.69816	0.89002	0.83973
W6	0.71126	0.84529	0.68322
O7	0.77883	0.84983	0.3479
O8	0.35389	0.71971	0.64002
O9	0.38763	0.94739	0.3528
O10	0.36853	0.83458	0.34937
O11	0.2912	0.79564	0.83523
O12	0.3025	0.88841	0.15736
W13	0.28847	0.84272	0.31269
O14	0.2213	0.85052	0.64542
O15	0.83121	0.37431	-0.00341
V16	0.68509	0.838	-0.00211
O17	0.69322	0.89088	0.49549
O18	0.7053	0.79341	0.49578
W19	0.61343	0.89368	0.48493
W20	0.62774	0.77062	0.50926
O21	0.55856	0.77352	0.51016
O22	0.54871	0.87262	0.50447
O23	0	0.69263	0.49518
O24	0.62824	0.37176	0.47691
O25	0.87305	0.12695	0.49769
O26	0.5	0.18153	0.73989
V27	0.81902	0.5	0.55976
V28	0.633	0	0.50991
V29	0.67056	0.67056	0.48082
V30	0.83056	0.83056	0.49752

Supplementary Table 19. Comparison of the framework porosity of the **VT** family with that of the existing zeolites.

material	porosity	material	porosity	material	porosity	material	porosity	material	porosity	material	porosity
RWY	71.28	AFX	47.04	IWR	42.77	CFI	38.23	LTJ	35.3	BIK	29.84
IRY	61.26	SFW	46.98	CON	42.69	ABW	38.21	BRE	35.28	BCT	29.75
CLO	60.23	FAU	46.86	MWW	42.05	STT	38.21	EZT	35.11	PCR	29.64
ITV	60.16	AFT	46.61	CSV	42.04	AFN	38.2	IFO	35.08	IHW	29.41
JSR	58.8	UFI	46.54	ATS	41.76	DOH	38.2	AWO	35.05	RWR	29.22
**VT-6**	58.38	VFI	46.52	ITE	41.75	VNI	38.17	FER	35.02	NSI	28.99
IRR	58.37	SAS	46.37	BOG	41.63	APC	38.13	ITR	34.93	AHT	28.96
**VT-5**	58.25	SFO	46.17	VSV	41.57	HEU	38.02	IMF	34.92	TON	28.76
**VT-10**	57.96	AFR	46.13	SOD	41.27	SSF	37.87	SVV	34.8	CAS	28.48
OBW	57.59	BEC	45.9	STI	41.12	LIO	37.81	RUT	34.73	MTT	27.72
BOZ	56.68	AVL	45.89	CAN	41.11	IFR	37.48	SFE	34.48	ATV	27.5
OSO	55.41	PAU	45.55	RTH	41.09	STF	37.45	TUN	34.34	CZP	26.9
IFU	54.78	IWS	45.54	RSN	41.07	MON	37.22	APD	34.33	OSL	26.41
NPT	54.64	NAB	45.49	LOV	40.8	MTN	37.02	PON	34.22	AEN	26.07
**VT-4**	54.62	THO	45.45	CGS	40.66	SEW	36.97	LAU	34.14	PAR	25.8
**VT-3**	54.38	ETR	45.43	LOS	40.65	NES	36.86	ETL	34.05	RON	23.68
TSC	54.33	MWF	44.97	SOS	40.56	UEI	36.85	MEL	34	AFO	23.63
**VT-9**	53.6	IWV	44.91	SAT	40.55	DAC	36.79	ITH	33.8	SAF	23.56
**VT-7**	52.48	SBN	44.79	MAZ	40.55	MAR	36.71	SSY	33.64	JNT	23.47
AFY	52.46	ISV	44.57	ATT	40.34	ITW	36.58	BOF	33.39	NPO	22.77
**VT-2**	52.34	ACO	44.39	LTF	40.3	JSN	36.58	CDO	33.21	ATO	22.53
SBE	52.09	EDL	44.39	UOV	40.2	EPL	36.55	IFY	33.09	VET	22.43
JST	51.98	GIS	44.03	ERL	40.11	UOS	36.46	JSW	33.03	GON	21.98
SBT	51.81	MER	44.01	OFF	40.1	TER	36.29	SVR	32.73	MTF	21.68
RHO	50.66	SIV	43.96	IFW	39.94	ESV	36.24	EEL	32.2	CHI	20.53
**VT-1**	50.45	AFV	43.96	AFG	39.89	TOL	36.17	SZR	32.12	MVY	19.98
SBS	49.41	FRA	43.95	EON	39.55	RRO	36.1	MTW	31.98	LIT	19.41
PUN	49.33	JOZ	43.87	OWE	39.32	BSV	36.03	JRY	31.93	PSL	12.93
MEL	49	PHL	43.86	SFH	39.29	LTL	35.89	MSO	31.92	MEP	0
SAO	48.98	POS	43.84	AWW	39.25	ZON	35.87	JBW	31.51	SGT	0
SAV	48.95	WEI	43.8	GIU	39.18	YUG	35.86	MFI	31.34		
**VT-8**	48.79	LTA	43.79	IWW	39.09	EUO	35.76	CGF	31.34		
BPH	48.36	USI	43.68	LTN	39.04	OKO	35.74	AFI	31.29		
GME	48.36	AST	43.47	SFN	38.87	SFF	35.68	NON	31.16		
CHA	48.32	STW	43.43	ITG	38.8	MFS	35.68	ANA	31		
AEI	48.22	EAB	43.17	UWY	38.78	MOZ	35.6	AET	30.85		
KFI	48.21	SOF	43	SFS	38.48	RTE	35.48	DON	30.76		
EMT	48.19	LEV	42.97	MSE	38.48	ATN	35.46	ASV	30.67		
AFS	48.02	IRN	42.86	DFT	38.43	DDR	35.44	UOZ	30.26		
DFO	47.14	UTL	42.81	MOR	38.23	GOO	35.33	SFG	30.17		

Supplementary Table 20. BVS of **VT-5** and corresponding structure**.**

	Bond length	BVS
W19-O	1.84	
	1.84	
	1.84	
	2.111	
	2.111	
	2.111	5.529
W5-O	1.84	
	1.84	
	1.845	
	2.109	
	2.098	
	2.087	5.577
W6-O	1.843	
	1.847	
	1.834	
	2.11	
	2.098	
	2.088	5.578
W21-O	1.845	
	1.845	
	1.845	
	2.112	
	2.112	
	2.112	5.474
V15-O	1.942	
	1.942	
	1.932	
	1.932	
	1.612	3.712
V13-O	1.931	
	1.923	
	1.923	
	1.92	
	1.583	3.907
V16-O	1.955	
	1.955	
	1.937	
	1.937	
	1.584	3.766

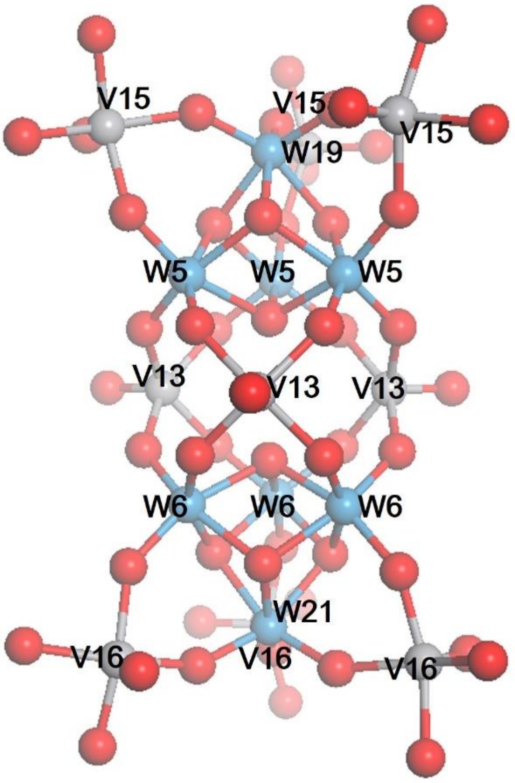
V15
V15
W19
V15
W5
W5
W5
V13
V13
V13
W6
W6
W6
W21
V16
V16
V16

Supplementary Table 21. Atomic positions of **VT-5** after Rietveld refinement and the corresponding structure.

Atom	*X*	*Y*	*Z*	U_{iso}	Occupancy
O1	0.27237	0.42033	0.68146	0.06	1
O2	0.61839	0.52742	0.19918	0.06	1
O3	0.61786	0.52527	0.31758	0.06	1
O4	0.27346	0.42201	0.80049	0.06	1
W5	0.4544	0.84165	0.63231	1E-3	1
W6	0.61139	0.1581	0.14969	1E-3	1
O7	0.38261	0.56154	0.47755	0.06	1
O8	0.2912	0.75934	0.57945	0.06	1
O9	0.42833	0.46559	0.57424	0.06	1
O10	0.55727	0.37844	0.99544	0.06	1
O11	0.75934	0.29133	0.09674	0.06	1
O12	0.46332	0.42628	0.09238	0.06	1
V13	0.25045	0.84978	0.74147	0.02	1
O14	0.20594	0.95028	0.74138	0.06	1
O15	0.41627	1.12974	0.85188	0.06	1
O16	0.11525	0.23213	0.75808	0.06	1
O17	0.41624	0.1298	0.85188	0.06	1
O18	0.48857	0.31962	0.74779	0.06	1
O19	0.35843	0.09963	0.65428	0.06	1
V20	0.48024	0	0.5	0.02	1
V21	0.55585	0	0	0.02	1
O22	0	0.3515	0.5	0.06	1
O23	0.31542	0.31542	0	0.06	1
W24	0.33333	0.66667	0.51122	1E-3	1
O25	0.33333	0.66667	0.67257	0.06	1
W26	0.33333	0.66667	0.97164	1E-3	1
O27	0.33333	0.66667	0.80992	0.06	1

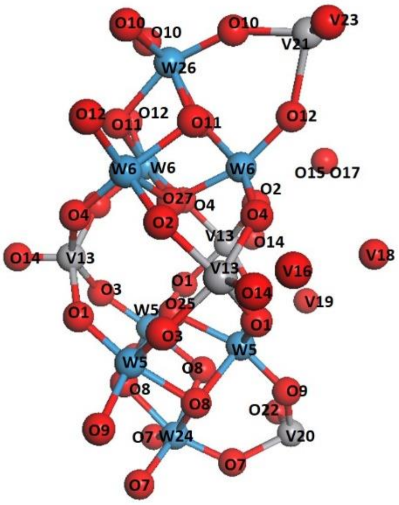

Supplementary Table 22. Results of the charge-flipping algorithm for **Cs-VT-1** and the corresponding structure.

Peaks	*X*	*Y*	*Z*	Intensity	Assignment
Q1	0.1706	0.3041	0.3116	27.14	W
Q2	0.1678	0.3322	0.6677	21.19	W
Q3	0.0018	0.48	0.2815	13.88	Cs
Q4	0.0005	0.4997	0.5003	5.68	Water
Q5	0.0632	0.5899	0.0748	5.84	Water
Q6	0.2046	0.7106	0.2027	6.58	O
Q7	0.026	0.2627	0.234	5.05	V
Q8	0.0736	0.3192	0.6891	5.85	O
Q9	0.2213	0.7213	0.7786	6.08	O
Q10	0.066	0.7445	0.7371	3.91	-

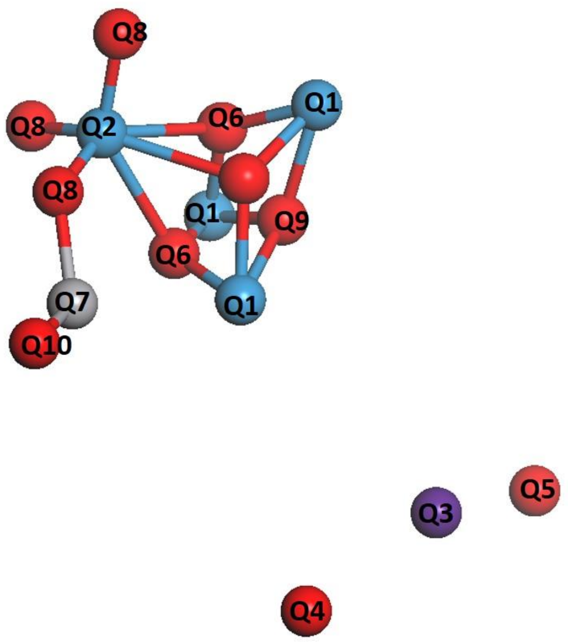

Supplementary Table 23. Atomic positions of **Cs-VT-1** after Rietveld refinement and the corresponding structure.

Atom	*X*	*Y*	*Z*	U_{iso}	Occupancy
W1	0.16941	0.69069	0.19356	0.01	1
Cs2	0.01408	0.51326	0.21197	0.01	0.98
V3	-0.00199	0.73938	0.27891	0.02	1
O4	0.04142	0.40277	0.4076	0.06	1
O5	0.1843	0.68207	0.32661	0.06	1
O6	0.07199	0.29934	0.81479	0.06	1
O7	0.30247	0.56912	0.32687	0.06	1
O8	0.29802	0.70519	0.44477	0.06	1
O9	0.18093	0.81095	0.45401	0.06	1
O10	0.40278	0.09243	0.54139	0.06	1
O11	0.47774	0.172	0.69078	0.06	1
O12	0.51644	0.332	0.84287	0.06	1
O13	0.78314	0.96648	0.97335	0.06	1
W14	0.16919	0.66919	0.83081	0.01	1
Cs15	0	0.5	0	0.01	0.48
O16	0.1991	0.3009	0.6991	0.06	1
O17	0	0.5	0.5	0.06	0.8

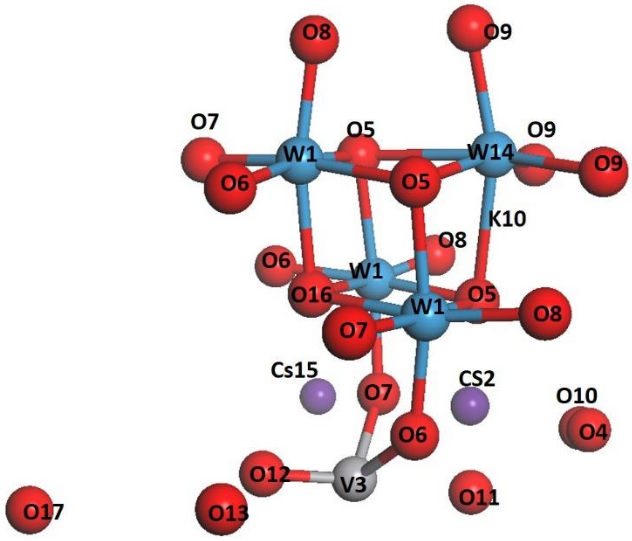

Supplementary Table 24. Number of small molecules per cubane unit adsorbed in the material.

	**VT-1**	**VT-5**
Unit	$[W_4V_3O_{19}]$	$[W_4V_3O_{19}]$
CH_4	1.1	-
C_2H_6	1.5	-
C_3H_8	1.3	-
n-C_4H_{10}	1.3	-
i-C_4H_{10}	0.4	-
Cyclohexane	0.4	-
CO_2	2.8	0.8
H_2O	10.0	7.6
CH_3OH	4.4	3.4
C_2H_5OH	2.9	2.5
Acetone	2.0	1.8

The value were calculated by the equation (1):

$$\text{Number of molecules} = \frac{[\text{adsorbed amount}](\text{cm}^3\ \text{g}^{-1}) \times [\text{molecular weight of the formula}]\ (\text{g mol}^{-1})}{22400\ (\text{cm}^3\text{mol}^{-1})} \qquad (1)$$

Supplementary Table 25. Comparison of **VT-1** and **VT-5** with other crystalline inorganic porous frameworks based on transition metal oxide and zeolites.

Material	Formula	Pore size	Blockage	Surface area (m^2 g^{-1})	Pore volume (cm^3 g^{-1})	The Largest adsorbed molecule
		Transition metal oxides and transition metal based oxides				
**VT-1**	$(NH_4)_{0.2}K_{1.5}H_{0.3}[W_4V_3O_{19}]$	4.3×4.3 Å	N	310	0.1	n-C_4H_{10}
**VT-5**	$[N(CH_3)_3]_{1.4}H_2[W_4V_3O_{19}]$	7.4 × 7.4 Å, 3.9 × 7.9 Å	P	-	-	Acetone
Trig MoVO	$Mo_3V_{1.15}O_x$	4.0×3.0 Å	Y	23.2	0.005	- [4]
Orth MoVO	$Mo_3V_{0.97}O_x$	4.0×3.0 Å	N	40.0	0.0137	C_2H_6 [4]
MoVBiO	$[Bi_2Mo_{9.4}V_{3.6}O_{40}]$	3.0×3.0 Å	N	60	0.02	C_2H_6 [5]
OMS-2		2.68 Å	Y	108	-	CO_2 [6]
LaMnO	$H[La(H_2O)_4]_2[MnV_{13}O_{38}]\cdot 9NMP\cdot 17H_2O$ (NMP= N-methyl-2-pyrrolidone)	21.54×13.76 Å, 22.69×6.38 Å	P	-	-	CH_3OH [7]
PMnWO	$K_{18}Li_6[Mn_8(H_2O)_{48}P_8W_{48}O_{184}]$	7.2×7.2 Å	P	-	-	NH_3 [8]
VBZnO	$Zn_6V_{10}B_{29}O_{85}$	24.7×12.7 Å 3.3×3.3 Å	P	143	-	CO_2 [9]
VPO	$Cs_{2.64}(V_5O_9)(AsO_4)_2$	6.9×6.9 Å	N	111	-	H_2O [10,11]
Pharmacosiderite	$HK_3Ti_4O_4(SiO_4)_3\cdot 4H_2O$	3.6×3.6 Å	Y	-	-	- [12]
		Zeolites				
LTA	-	4.21×4.21 Å	N	599	0.23	- [13]
BETA	-	5.95×5.95 Å	N	450	0.18	- [14]
MFI	-	4.7×4.46 Å	N	329.5	0.151	- [15]
FAU	-	7.35×7.35 Å	N	539	0.327	- [16]
MOR	-	6.45 Å	N	448	0.1633	- [17]

Supplementary Table 26. The relationship between the temperature and the vapour pressure of water when capillary condensation (pore filling) occurred estimated by the Kelvin equation.

Temperature (^{o}C)	ρ (kg m^{-3}) [18]	σ (N m^{-1}) [19]	p_0 (kPa) [20]	p (kPa)	*Vol*% [a]
150	916.1287819	0.04874	472.5519086	92.01	90.80
140	925.3326778	0.05085	358.9651729	63.57	62.74
130	934.0911806	0.05293	268.706703	43.14	42.57
120	942.4097092	0.05496	197.9717544	28.72	28.34
110	950.2885172	0.05696	143.3647696	18.72	18.47
100	957.7223216	0.05891	101.8929744	11.92	11.77
90	964.6997829	0.06082	70.95431039	7.41	7.31
80	971.2027609	0.06267	48.3199255	4.48	4.42
70	977.2052245	0.06447	32.11164611	2.63	2.60
60	982.6716125	0.06624	20.77505759	1.49	1.47
50	987.5542946	0.06794	13.04901599	0.82	0.81
40	991.7894952	0.0696	7.932584148	0.43	0.42
30	995.2904755	0.0712	4.650516236	0.22	0.21

[a] volume percentage in 101.325 kPa.

Supplementary Table 27. The relationship between the temperature and the vapour pressure of NH_3 when capillary condensation (pore filling) occurred estimated by the Kelvin equation.

Temperature (°C)	ρ (kg m^{-3}) [21]	σ (N m^{-1}) [22, a]	p_0 (kPa) [23, b]	p (kPa)	*Vol*%
150	485.8	-	15067.64774	-	-
140	497.5	0.00129	12821.25888	11869.4	-
130	509.8	0.00322	10840.44541	8941.6	-
120	522.8	0.00452	9101.395264	6945.7	-
110	536.4	0.00548	7582.092755	5463.3	-
100	550.9	0.00709	6262.159055	4098.4	-
90	566.2	0.0071	5122.695184	3351.0	-
80	582.4	0.00871	4146.130839	2463.7	-
70	599.7	0.01064	3316.082462	1756.4	-
60	618.2	0.01258	2617.224105	1235.3	-
50	638	0.01484	2035.17472	840.2	-
40	659.3	0.01677	1556.405529	573.5	-
30	682.2	0.01903	1168.170854	377.0	-

[a] The data is from theoretical equation at 100-140 °C. [b] The data is out of range of the equation at 80-140 °C.

Supplementary Discussion

The Kelvin equation [24] was used to estimate physical adsorption of water and NH_3 and their competition.

$$\ln\frac{p}{p_0} = \frac{-2\sigma M}{RT\rho r} \qquad (2)$$

In this equation, p is the vapour pressure of the liquid for capillary condensation (pore filling). p_0 is the saturated pressure of the adsorbate. σ is the surface tension of the adsorbate. M is the molar weight of the adsorbate. R is the ideal gas constant. T is temperature. ρ is the density of the adsorbate, r is radius of curvature when pore filling occurs.

R and M are constants. σ, ρ, and p_0 are temperature dependent values, which are obtained or estimated according to the literatures at different temperatures (Supplementary Table 26 and Table 27). r is determined using the experimental water adsorption isotherm (Figure 5e in main text) at 25 ^{o}C by the Kelvin equation and proposed to be constant for water and NH_3. $r = 3.32710\times10^{-10}$ m.

Having the above information we estimated the vapour pressure (p) for capillary condensation (pore filling) at different temperatures. When the water content is 11.77%-18.47%, the condensation temperature in the micropore of **VT-1** is 100-110 ^{o}C (Supplementary Table 26). When the water content is 1.47%-2.60%, the condensation temperature is 60-70 ^{o}C. The vapour pressure (p) for capillary condensation (pore filling) of NH_3 is higher than that of water. Therefore, water preferentially adsorbs in **VT-1**, and water would inhibit the reaction of **VT-1**.

The Kelvin equation estimation is only based on physical adsorption. NH_3 also has chemical interaction with **VT-1**. NH_3-TPD clearly shows that **VT-1** adsorbs NH_3 at 100 ^{o}C, corresponding to the chemical adsorption (supplementary Figure 20). Chemical adsorption is usually slower but stronger than physical adsorption. Therefore, water is likely to be adsorbed in **VT-1** rapidly, but still a part of NH_3 can be adsorbed and reacted with NO.

Supplementary Reference:

1. Ffenberger, H. E., Iester, G. G. & Rause, W. K. Tschortnerite, a copper-bearing zeolite from the Bellberg volcano, Eifel, Germany. *Am. Mineral.* **83,** 607–617 (1998).

2. Oleksiienko, O., Wolkersdorfer, C. & Sillanpää, M. Titanosilicates in cation adsorption and cation exchange – A review. *Chem. Eng. J.* **317,** 570–585 (2017).

3. Anderson, M. W. *et al.* Structure of the microporous titanosilicate ETS-10. *Nature* **367,** 347–351 (1994).

4. Chen, C., Kosuke, N., Murayama, T. & Ueda, W. Single-crystalline-phase Mo_3VO_x: an efficient catalyst for the partial oxidation of acrolein to acrylic acid. *ChemCatChem* **5,** 2869–2873 (2013).

5. Zhang, Z. *et al.* Tetrahedral connection of ε-Keggin-type polyoxometalates to form an all-Inorganic octahedral molecular sieve with an intrinsic 3D pore system. *Inorg. Chem.* **53,** 903–911 (2014).

6. Espinal, L. *et al.* Time-dependent CO_2 sorption hysteresis in a one-dimensional microporous octahedral molecular sieve. *J. Am. Chem. Soc.* **134,** 7944–7951 (2012).

7. Liu, D. *et al.* Polyoxometalate-based purely inorganic porous frameworks with selective adsorption and oxidative catalysis functionalities. *Chem. Commun.* 3673–3675 (2013).

8. Zhan, C. *et al.* A metamorphic inorganic framework that can be switched between eight single-crystalline states. *Nat. Commun.* **8,** 14185 (2017).

9. Chen, H. *et al.* Construction of mesoporous frameworks with vanadoborate clusters. *Angew. Chem. Int. Ed.* **53,** 3608–3611 (2014).

10. Queen, W. L., Hwu, S.-J. & Reighard, S. Salt-templated mesoporous solids comprised of interlinked polyoxovanadate clusters. *Inorg. Chem.* **49,** 1316–1318 (2010).

11. Khan, M. I. *et al.* Giant voids in the hydrothermally synthesized microporous square pyramidal-tetrahedral framework vanadium phosphates $[HN(CH_2CH_2)_3NH]K_{1.35}[V_5O_9(PO_4)_2]xH_2O$ and $Cs_3[V_5O_9(PO_4)_2]xH_2O$. *Chem. Mater.* **8,** 43–53 (1996).

12. Lopes, C. B., Coimbra, J., Otero, M., Pereira, E. & Duarte, A. C. Uptake of Hg^{2+} from aqueous solutions by microporous titano-and zircono-silicates. *Quim. Noca* **31,** 321–325 (2008).

13. Zhao, X. *et al.* Highly efficient synthesis of LTA-type aluminophosphate molecular sieve by improved ionothermal method. *New J. Chem.* **40,** 2444–2450 (2016).

14. Zhang, H. *et al.* Rational synthesis of Beta zeolite with improved quality by decreasing crystallization temperature in organotemplate-free route. *Microporous Mesoporous Mater.* **180,** 123–129 (2013).

15. Yang, Q. *et al.* Aluminum fluoride modified HZSM-5 zeolite with superior performance in synthesis of dimethyl ether from methanol. *Energy & Fuels* **26,** 4475–4480 (2012).

16. Godhani, D. R., Nakum, H. D., Parmar, D. K., Mehta, J. P. & Desai, N. C. Zeolite-Y immobilized metallo-ligand complexes : a novel heterogenous catalysts for selective oxidation. *Inorg. Chem. Commun.* **72,** 105–116 (2016).

17. Reule, A. A. C., Sawada, J. A. & Semagina, N. Effect of selective 4-membered ring dealumination on mordenite-catalyzed dimethyl ether carbonylation. *J. Catal.* **349,** 98–109 (2017).

18. Jones, F. E. & Harris, G. L. ITS-90 Density of water formulation for volumetric standards calibration. *J. Res. Natl. Inst. Stand. Technol.* **97,** 335–340 (1992).

19. Vargaftik, N. B., Volkov, B. N. & Voljak, L. D. International Tables of the Surface Tension of Water. *J. Phys. Chem. Ref. Data* **12,** 817–820 (1983).

20. Antoine, C. Tensions des vapeurs: nouvelle relation entre les tensions et les temperatures. *Compt. Rend. Acad. Sci.* **107,** 681–684, 778–780, 836–837. (1888).

21. Engineering ToolBox, (2018). Ammonia-density at varying temperature and pressure. [online] Available at: https://www.engineeringtoolbox.com/ammonia-density-temperature-pressure-d_2006.html.

22. Gloor, G. J. *et al.* An accurate density functional theory for the vapor-liquid interface of associating chain molecules based on the statistical associating fluid theory for potentials of variable range. *J. Chem. Phys.* **121,** 12740–12759 (2004).

23. Cragoe, C. S., Meyers, C. H. & Taylor, C. S. The vapor pressure of ammonia. *J. Am. Chem. Soc.* **42,** 206–229 (1920).

24. Yin, G., Liu, Q., Liu, Z. & Wu, W. Extension of Kelvin equation to CO_2 adsorption in activated carbon. *Fuel Process. Technol.* **174,** 118–122 (2018).
